# Immune crosstalk in metabolic dysfunction-associated steatotic liver disease: interactions between innate and adaptive immunity

**DOI:** 10.3389/fimmu.2026.1835197

**Published:** 2026-06-29

**Authors:** Xiangqiong Li, Kunnu Jin, Jindong Fang, Xuan Ma, Jiamei He, Ancheng Lu, Zhuangzhuang Jia, Anhua Shi

**Affiliations:** 1 Collge of Basic Medical Sciences, Yunnan University of Chinese Medicine, Kunming, Yunnan, China; 2 Yunnan Key Laboratory of Integrated Traditional Chinese and Western Medicine for Chronic Disease in Prevention and Treatment, Kunming, Yunnan, China; 3 Key Laboratory of Microcosmic Syndrome Differentiation, Education Department of Yunnan, Kunming, Yunnan, China

**Keywords:** adaptive immunity, immune cell, immunotherapy, innate immunity, MASLD

## Abstract

Metabolic dysfunction-associated steatotic liver disease (MASLD) has emerged as the leading chronic liver disorder worldwide. Its pathological progression typically begins with hepatic steatosis, advances to metabolic dysfunction-associated steatohepatitis (MASH) and fibrosis, and in some cases culminates in hepatocellular carcinoma (HCC). This continuum is influenced not only by metabolic disturbances but also by coordinated local and systemic inflammatory and immune responses. This article systematically summarizes the inflammatory mechanisms underlying MASLD/MASH progression, highlighting their origin in the dynamic crosstalk between innate and adaptive immune compartments. Such interactions involve multiple immune cell subsets, including macrophages, T lymphocytes, and B lymphocytes, and are governed by intricate molecular regulatory pathways. Furthermore, this review explores how these interconnected processes amplify hepatic inflammation and fibrogenesis and discusses potential therapeutic approaches to modulate key nodes within immune signaling networks.

## 1 Introduction

**Figure 1:**
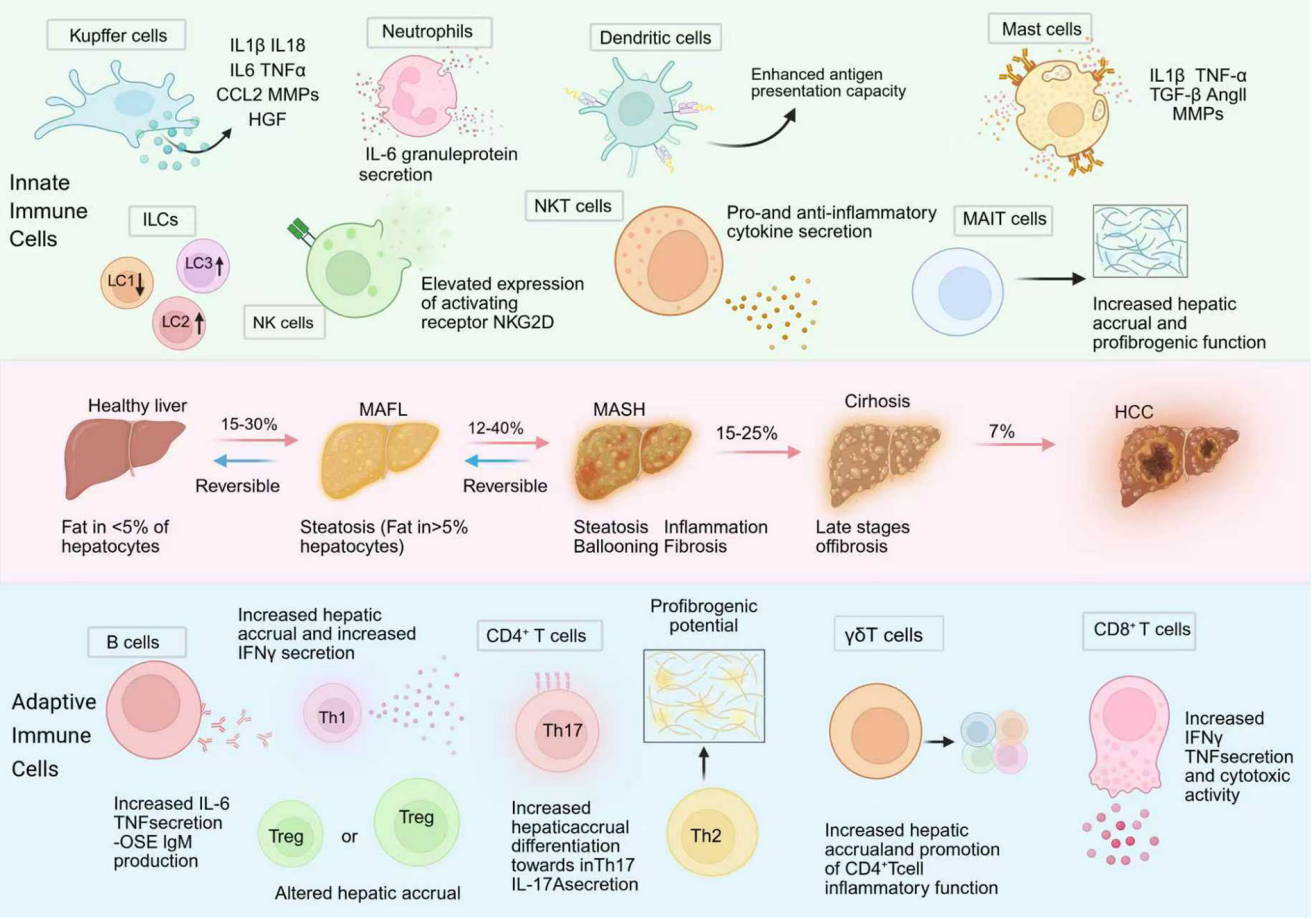
Immunological landscape of MASLD pathogenesis. During the development of MASLD, the composition and function of liver immune cells will change from the healthy liver, reversible stage, MASH, to cirrhosis, and the estimated transition risks are 15-30%, 12-40%, 15-25%, and 7%, respectively. In the innate immune part, Kupffer cells secrete IL1β, IL-18, IL-6, TNF-α, CCL2, MMPs, and HGF. The antigen-presenting ability of neutrophils and dendritic cells was enhanced. Mast cells release IL-1 β, TNF α, TGF β, AngII, and MMPs, which promote fibroinflammatory remodeling. In innate lymphocytes, LC1 decreased, while LC2 and LC3 increased. NK cells up-regulate NKG2D, while NKT cells have both pro-inflammatory and anti-inflammatory cytokine characteristics. MAIT cells accumulate in the liver and acquire the characteristics of promoting fibrosis. In the part of adaptive immunity, B cells gather in the liver, and IFN-γ secretion increases. CD4^+^ T cells have the potential to promote fibrosis. Th1 cells promote the differentiation of liver into Th17 and secretion of IL-17a, while Th17 cells aggravate the inflammatory reaction of CD4^+^ T cells in the liver. Th2 cells also accumulate in the liver and promote the inflammatory response driven by CD4^+^ T cells. Image was partly created with https://biorender.com/. IL-1β, Interleukin-1 beta; IL-18, Interleukin-18; IL-6, Interleukin-6; TNF-α, Tumor necrosis factor alpha; MMPs, Matrix metalloproteinases; HGF, Hepatocyte growth factor; NKG2D, Natural killer group 2 member D.

Metabolic dysfunction-associated steatotic liver disease (MASLD) encompasses the full spectrum of this condition, ranging from the early stage of metabolic dysfunction-associated steatotic liver (MASL) to the more advanced form, metabolic dysfunction-associated steatohepatitis (MASH) (1). MASL is characterized by hepatic triglyceride accumulation and is generally considered reversible, accompanied by mild inflammation. MASH, however, is marked by lobular inflammation, fibrosis, and hepatocellular ballooning, which may progress to irreversible cirrhosis and potentially lead to hepatocellular carcinoma (HCC) (2–4). This nomenclature, now widely endorsed by major liver societies, supersedes the prior terminology of non-alcoholic fatty liver disease (NAFLD) (5). MASLD has emerged as a leading cause of chronic liver disease globally and is becoming a predominant indication for liver transplantation due to its complications of cirrhosis and HCC (6). Approximately one out of every four individuals worldwide is impacted by this condition, and it’s closely associated with obesity, insulin resistance, diabetes, and dyslipidemia (7). Currently, MASLD affects 38% of adults and 7-14% of teenagers. It is estimated that by 2040, the prevalence rate of adults will exceed 55%. Mortality patterns vary according to disease stage: patients with liver cirrhosis are mainly liver problems, while those without liver cirrhosis are more prone to cardiovascular diseases and extrahepatic malignancies (8). At present, the mainstream model for explaining the onset of MASLD is the “multiple-hit hypothesis” (9). Initially, liver fat accumulation will make the liver more vulnerable to secondary injury, such as lipotoxicity, oxidative stress, gut dysbiosis, and pro-inflammatory factors, indicating that MASLD is a systemic inflammatory disorder (10). Innate immune activation contributes to MASL, but the requirement of adaptive immunity for MASH progression is context-dependent. Some adaptive subsets may even exert protective functions (11) (**Figure 1**). Traditionally, innate and adaptive immunity were considered to function sequentially, but recent research found that they actually have bidirectional crosstalk between innate and adaptive immunity, and this interaction was very important (12). This review studies the complex cellular and molecular interactions between innate immunity and adaptive immunity in MASLD, and also evaluates some emerging immunotherapies for these pathways.

## 2 Innate immune cells

This section classifies innate immune cells based on their primary functional roles in MASLD into two groups: phagocytic and cytotoxic innate immune cells and regulatory and tissue-modulating innate immune cells, to clearly reflect their biological functions. The innate immune system is an evolutionarily conserved first-line defense, and many of its fundamental features are highly conserved across species. It functions in almost all tissues, including hematopoietic cells and non-hematopoietic cells (13). Activation of these cells results in dysregulated production of pro-inflammatory cytokines and causes the immune cells in the liver to progressively accumulate. Subsequently, inflammation and cell damage in the liver are more severe (14). In MASLD, immune cells play a central role in the inflammatory reaction, damage, and hepatic fibrosis. They can recognize danger-associated molecular patterns (DAMPs) and then initiate rapid immune responses.

### 2.1 Phagocytic and cytotoxic innate immune cells

#### 2.1.1 Macrophage

Macrophages are innate immune cells with potent phagocytic capacity. Macrophages are present in almost all tissue types from embryonic development through adulthood in the human body. These cells are particularly adaptable to the environment and will change their functions, shapes, and phenotypes in response to local microenvironmental cues and systemic signals (15). When the immune system is activated, macrophages mediate rapid innate immune defense, coordinate an antigen-specific adaptive immune response, and recruit additional immune cells. Besides phagocytosis, macrophages secrete a variety of cytokines, chemokines, and growth factors, which are critical in host defense as well as in tissue repair. Accumulating evidence has highlighted the role of macrophages in metabolic diseases, especially insulin resistance and type 2 diabetes mellitus (T2DM) (16–18). Macrophages are broadly categorized into two primary polarization states: classically activated (M1) and alternatively activated (M2). In immune responses, M1 macrophages promote Th1 reactions through the production of pro-inflammatory cytokines. M2 macrophages produce elevated interleukin-10(IL-10) and transforming growth factor beta (TGF-β) but minimal interleukin-12 (IL-12) and interleukin-23(IL-23). These cells drive Th2-mediated processes, including tissue repair, inflammation resolution, allergic responses, and immune modulation (19). In disease settings, the traditional binary view of pro-inflammatory M1 and reparative M2 states has thus been supplanted by a more dynamic continuum model. The liver-specific macrophages are called Kupffer cells (KCs), which are a resident macrophage population in this organ.

**Figure 2:**
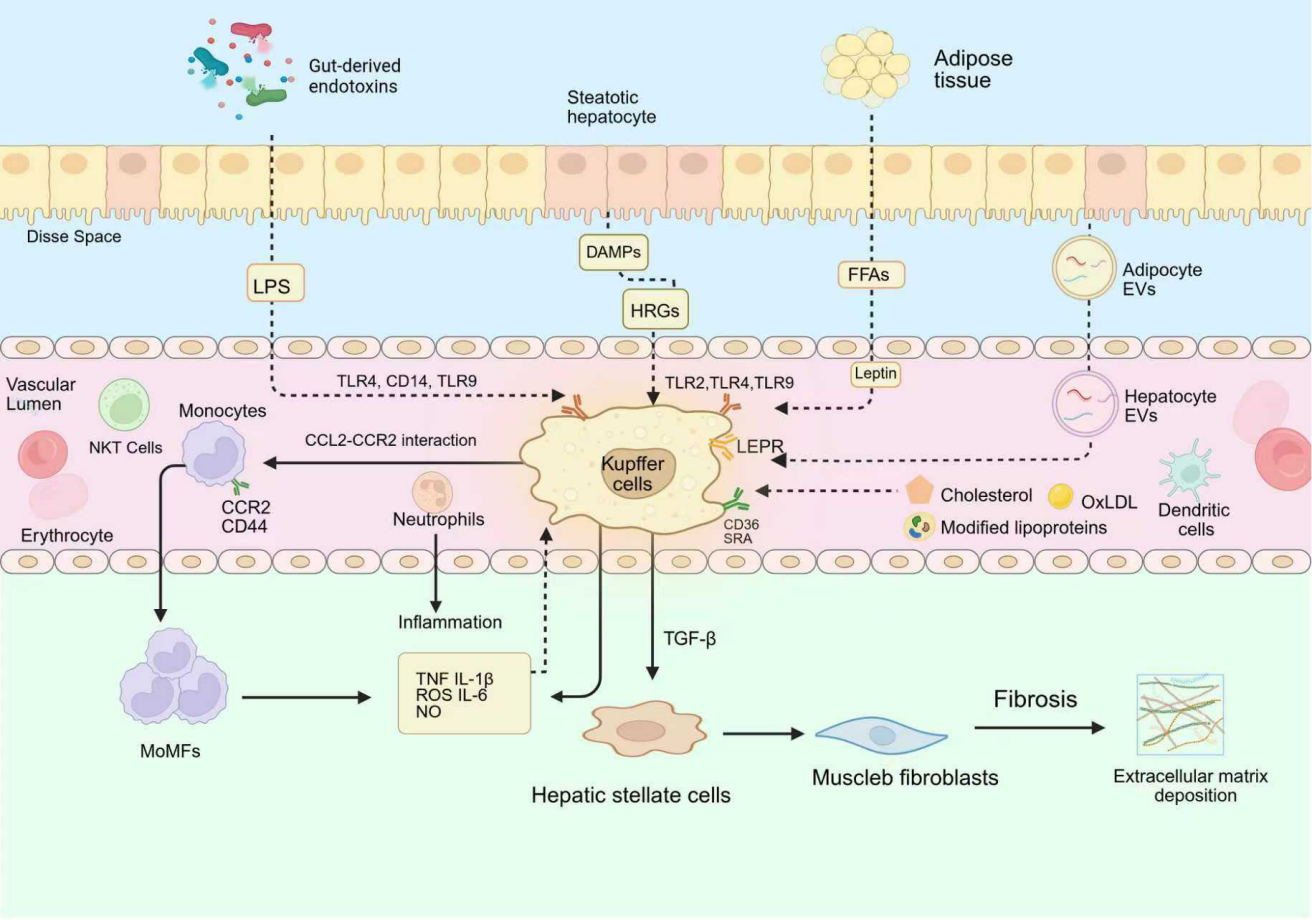
Kupffer cell activation and effector pathways in MASLD. Several different signals will converge at Kupffer cells in the lumen-luminal space. Intestinal endotoxin, such as LPS, will act through TLR4, CD14, and TLR9. The adipose tissue secretes the FFAs and leptin, which is mediated by LEPR. Fatty liver cells release DAMPS, HRG EVs. Cholesterol, modified lipoproteins, and oxidized low-density lipoproteins oxldl are recognized by SRA and CD36. These signals together make Kupffer cells produce TNF, IL-1β, IL-6, ROS and NO. Activated Kupffer cells also secrete CCL2, which can call monocytes and neutrophils from the blood through CCR2. MoMacs can make liver inflammation worse and provide TGF-β together with Kupffer cells. TGF-β can turn hepatic stellate cells into myofibroblasts that can secrete matrix, and eventually lead to extracellular matrix accumulation and fibrosis. There are dendritic cells, NKT cells, and red blood cells in the vascular lumen, but their specific mechanism of action in this schematic diagram is not fully understood. Image was partly created with https://biorender.com/. LPS, lipopolysaccharide; TLR4, Toll-like receptor 4; LEPR, leptin receptor; DAMPS, Danger-associated molecular patterns; FFAs, free fatty acids; HRG, histidine-rich glycoproteins; oxldl, oxidized low-density lipoproteins; SRA, scavenger receptor A; ROS, reactive oxygen species; EVs, extracellular vesicles; MoMacs, Monocyte-derived macrophages.

In sterile inflammation, KCs, the resident macrophages of the liver, facilitate tissue regeneration by clearing extracellular matrix (ECM) and cellular debris. In MASH, however, these cells can perpetuate inflammation and fibrosis by releasing mediators such as TGF-β1 and interleukin-6 (IL-6) (20). KCs reside in the hepatic sinusoids, and they serve as key immune sentinels (**Figure 2**). They initially generate pro-inflammatory agents like tumor necrosis factor-alpha (TNF-α) and CCL2, thereby promoting steatohepatitis. In the early stage of MASH, KC activation can help reduce inflammation and liver damage, which shows that they play a role at the beginning of the disease. At the cellular level, free fatty acids (FFAs) induce mitochondrial damage and mtDNA release, thereby activating the NLRP3 inflammasome and promoting IL-1β secretion in hepatocytes. Both animal and human studies have demonstrated increased NLRP3 inflammasome activation and elevated IL-1β levels during MASLD progression (21). Rodent studies using dietary MASH models have shown that systemic depletion of KCs and macrophages with clodronate liposomes or gadolinium chloride prevents steatosis, necroinflammation, and fibrosis. KCs promote hepatic lipid accumulation by suppressing peroxisome proliferator-activated receptor alpha activity via an IL-1β-dependent mechanism. Moreover, KCs -produced TNF-α can inhibit fatty acid oxidation and augment triglyceride accumulation. In Co-culture experiments(*in vitro*) involving KCs and hepatocytes demonstrated that TNF-α neutralization attenuated hepatic lipid accumulation (22). In addition to resident Kupffer cells, the role of monocyte-derived macrophages (MDMs) in the progression. In addition to resident Kupffer cells, monocyte-derived macrophages (MDMs) play important roles in MASLD progression. Single-cell RNA sequencing revealed that infiltrating monocytes differentiate into monocyte-derived Kupffer cells (MoKCs) and lipid-associated macrophages (LAMs) characterized by high CD9 and TREM2 expression (23). The identification of LAMs has fundamentally shifted the understanding of macrophage biology in MASLD, moving beyond the classical pro-inflammatory M1 versus reparative M2 dichotomy toward a more nuanced, stage-dependent functional paradigm. A recent spatial multi-omics study of 61 human livers (10 controls, 17 MASL, 34 MASH) by Li and colleagues provided critical insights into the functional heterogeneity of LAMs in MASLD (24). This study identified microphthalmia-associated transcription factor (MITF) as a key regulator of the lipid-handling capacity of LAMs, with MITF activity selectively elevated in LAMs from MASH patients. Functional experiments confirmed that MITF overexpression upregulates key LAM markers and genes involved in fatty acid oxidation via the PGC1α-PPARγ axis. Beyond lipid metabolism, the same study revealed an unexpected hepatoprotective role of LAMs: LAMs secrete hepatocyte growth factor (HGF), which activates MET receptors on hepatocytes, thereby promoting hepatocyte proliferation and reducing lipotoxicity-induced apoptosis. These findings challenge the unidirectional narrative that macrophages are merely pro-inflammatory effectors, positioning LAMs as a functionally heterogeneous population with both lipid-clearing and tissue-repair capacities. Under MASLD conditions, LAMs accumulate in steatotic regions, including portal, periportal, and midlobular areas (25). Spatial proteogenomic analyses further showed reduced IL-1β and TNF-α expression in LAMs following prolonged Western diet feeding (26). In MASH mouse models transplanted with Trem2-deficient bone marrow, pro-inflammatory cytokines, chemokines, and inflammasome-associated genes were upregulated, whereas anti-inflammatory genes were suppressed (27) Elevated plasma TREM2 levels in cirrhotic patients correlate with higher NAS scores and liver stiffness, suggesting that scar-associated macrophages (SAMs) may represent a profibrotic LAM subset characterized by Spp1 upregulation (28). In MASLD mouse models, THRSP-mediated MIF secretion recruits CD74+ LAMs and promotes hepatocyte–macrophage crosstalk, while the THRSP–MIF–CD74 axis has been proposed as a therapeutic target in MASH (29). In TREM2−deficient mouse models, impaired toxic lipid clearance by macrophages aggravates hepatocyte apoptosis, inflammation, and fibrosis. In addition, excessive Activin expression suppresses the LAM marker Gpnmb, whereas Gpnmb knockdown reproduces Activin A-induced transcriptional alterations in MASLD (30). CD11c+ and Mincle+ macrophages form crown-like structures that interact with dying hepatocytes and promote fibrosis, whereas Egr2 deficiency reduces CD11chiLy6Chi LAM accumulation and attenuates fibrosis (31, 32). In hepatocellular carcinoma models, LAMs exhibit immunosuppressive and tumor-promoting properties Moreover, a profibrotic Ceacam1+Msr1+Ly6C−F4/80−Mac1+ atypical monocyte population has been identified in murine liver fibrosis models. Consistently, constitutive TREM2 deficiency accelerates MASH progression, and Trem2-/- macrophage-derived exosomes may further disrupt hepatocyte mitochondrial function (33–35).

#### 2.1.2 Neutrophils

Neutrophils are the most abundant circulating leukocytes, and they are the most important in the pathophysiology of chronic inflammation (36). These cells release proteases such as matrix metalloproteinases and neutrophil elastase, and they will also produce an oxidative burst that destroys cell membranes, thus damaging tissues. Neutrophils also form neutrophil extracellular traps (NETs), which are a network structure composed of DNA, histones, and inflammatory proteins, which promote cancer development and chronic inflammation. Although NETs do not directly harm hepatocytes, they can accelerate blood coagulation, increase the formation of fibrin, and make the pro-inflammatory environment of the liver more serious. Even if it is not directly pathogenic, NETs influence the inflammatory pattern of the liver by activating macrophages and regulating T cell activity (37). Clinical studies have shown that MASLD patients have high levels of NETs formation biomarkers, and these levels correlate positively with disease severity. NETs were identified in early-stage MASH mouse models, where DNase administration reduced disease severity and slowed hepatocellular carcinoma progression. In mouse models and *in vitro* T−cell culture experiments, elevated NET levels are associated with an increase in hepatic regulatory T cells. NETs modulate transcriptional programs in immature CD4+ T cells to enhance Treg function and influence MASH progression (38). In preclinical studies (animal models and cell-based experiments), NETs drive metabolic reprogramming of hematopoietic stem cells via the Toll-like receptor 3/cyclooxygenase-2 pathway, contributing significantly to MASH-related liver fibrosis. Human neutrophil peptide 1 (HNP 1) promotes MASH pathogenesis by activating Kupffer cells, recruiting and activating macrophages, expanding activated hepatic stellate cells (HSCs), and driving hepatic inflammation and fibrosis (39). Clinical studies (human evidence), serum levels of myeloperoxidase DNA (MPO DNA), a NETs marker, are higher in MASH patients than in healthy individuals. A recent study further confirmed that markers of NETs formation, such as citrullinated histone H3, are elevated not only in serum but also directly in the liver tissue of MASH patients, and their levels correlate with MASLD severity (40).

In mouse models of MASLD, neutrophil−derived miR−223 inhibits disease advancement via immune modulation. miR−223 deficiency exacerbates inflammation and promotes fatty liver disease progression from steatosis to cancer (41). Neutrophil-derived matrix metalloproteinase-9 (MMP9) is essential for collagenase activity and promotes collagen resorption during liver repair, facilitating subsequent hepatocyte-driven MMP-mediated regeneration (42). Compared with obese patients, there are more elevated levels of CXCL1, IL-8, and E-selectin in the liver of patients with MASH, which will attract neutrophils. Moreover, their blood contains elevated levels of neutrophil elastases, which indicates that MASLD may be more serious (43). Research has found that elevated levels of CXCL1 in the liver of mice fed a high-fat diet (HFD) accelerate the progression of MASH. This is because CXCL1 activates stress kinases and allows neutrophils to release reactive oxygen species (ROS). These neutrophils, attracted by CXCL1, will produce more ROS and start stress kinases such as p38MAPK and apoptosis signal-regulating kinase 1(ASK1). Then, these kinases will transmit oxidative stress to downstream signals, which will eventually lead to cell death (44–46). A feedforward signal loop will make neutrophils live longer, which will aggravate liver inflammation and fibrosis. ROS produced by activated neutrophils can also activate HSC. In human MASH patients, the neutrophil−to−lymphocyte ratio is closely associated with MASLD severity, particularly reflecting the severity of lobular inflammation, fibrosis, and hepatocellular ballooning. Recently, another study found that this ratio is related to hepatocyte degeneration, steatosis, inflammation, fibrosis, and MASLD activity score, indicating that it may be a marker to judge the histological grading and fibrosis stage of MASH (47).

#### 2.1.3 Natural killer T cells

Natural Killer T (NKT) cells account for nearly one-third of hepatic lymphocytes. They are unique lymphocytes with both T cell receptors (TCRs) and NK cell receptors. Like NK cells and cytotoxic T lymphocytes (CTLs), these cells can destroy malignant targets through cytotoxic mechanisms (48). In the mouse liver, there are about four times more NKT cells than NK cells, but in the human liver, there are more NK cells. The activation state of NKT cells is very important for immune function, including tumor surveillance, autoimmune responses, and pathogen clearance. NKT cells are mainly divided into two types: type I and type II. Type I NKT cells, also known as invariant natural killer T (iNKT) cells, possess a semi-invariant TCR α chain and are specialized in recognizing lipid antigens presented by CD1d. In mice, iNKT cells account for up to half of hepatic lymphocytes, whereas the type II subset predominates in human livers (49).

MASLD liver samples, NKT cell counts decline. Mechanistic studies in mouse models indicate that this phenomenon involves reduced CD1d expression under endothelial stress and lipid presentation, while Kupffer cells promote NKT cell death and secrete IL−12. IL-15 production by KCs, which stimulates NKT cells, is reduced in MASLD (50). Conversely, some human studies have reported higher NKT cell numbers in advanced MASLD, possibly as a result of improved functional activation of KCs via CD1d-dependent pathways (51). MASH is driven by CD1d-lipid-antigen recognition, activating NKT cells. Depletion of KCs reduces hepatic IL-12 expression and restores NKT cell numbers in choline-deficient mice. Furthermore, an HFD-induced fatty liver increases KC numbers and enhances proinflammatory cytokine expression, causing NKT cell hyperactivation and death, which results in the depletion of hepatic NKT cells as MASLD progresses. Mice with MASH caused by the methionine-choline-deficient (MCD) diet showed an accumulation of hepatic NKT cells. MASH progression involves NKT cells that promote hepatic inflammation and steatosis via cytokines upon lipid antigen recognition. In mouse models, NKT cells promote fibrosis by secreting OPN and Hedgehog ligands via IL−15−activated Hedgehog signaling in HSCs. In contrast to wild-type mice consuming an HFD, those minus the IL-15 or IL-15Rα gene deficiency showed fewer hepatic CD4+, CD8+, and NKT cells, not to mention a lesser degree of steatosis and lobular inflammation (52). Research indicates that tumor necrosis factor superfamily member 14 (TNFSF14), a molecule that interacts with the lymphokine β receptor (LTβR), is produced by liver-resident NKT cells. When this substance connects with LTβR on liver cells, it triggers the nuclear factor kappa-B (NF-κB) signaling pathway and acts in concert with CD8+ T cells to promote tumor progression (53). Some mouse model studies suggest that NKT cells attenuate fat accumulation, adipose tissue inflammation, and insulin resistance. However, other studies (in both humans and mice) have reported contradictory results, with some even showing worsened outcomes (54). Previous studies suggest that the interleukin-4/Signal Transducer and Activator of Transcription 6(IL-4/STAT6) signaling pathway enables NKT cells’ beneficial impacts on glucose regulation (55). In both human and mouse MASLD, the reduction in hepatic NKT cells results from Tim−3−mediated apoptosis in terminally differentiated T cells. Although Tim-3’s natural ligand, galectin-9, decreased liver fat and mouse weight, it paradoxically expanded Tim-3-negative NKT cells through KCs and IL-15 involvement (56). Different NKT cell subsets participate in the regulation of disease progression, forming a complex regulatory network. NKT cell numbers decrease in both human and mouse MASLD studies. This decrease is related to the increase of apoptosis induced by IL-12 produced by KC and the enhancement of Tim-3/Gal-9 signal. In the MASH model, Jα18 and CD1d KO mice lacking NKT cells gained more weight and had more severe liver steatosis (57). The role of NKT cells in MASLD is context-dependent, varying by subset, activation state, disease stage, and local cytokine milieu. Thus, no unidirectional conclusion applies universally.

### 2.2 Regulatory and tissue-modulating innate immune cells

#### 2.2.1 Liver sinusoidal endothelial cells

Liver sinusoidal endothelial cells (LSECs) are specialized endothelial cells that are located between the blood and liver tissue and directly contact hepatocytes. The hepatic lobules are separated by the Disse space and the thin-walled hepatic sinusoids, which contain LSECs, and there are HSCs in them. In normal conditions, LSECs maintain hepatic homeostasis (58). LSECs exhibit anti-inflammatory and anti-fibrotic properties by preventing the activation of KCs and HSCs, thereby regulating hepatic vascular resistance and portal pressure. Early in MASLD, LSECs undergo capillarization, losing their fenestrae, and show impaired vasodilator production (59). Mice on a choline-deficient, L-amino acid-defined diet showed sinusoidal thinning within a week, while rats fed an HFD exhibited similar changes after three weeks (60). Even in the absence of significant inflammation or HSC activation, in the early stage of MASLD, LSECs undergo capillarization. Abnormal LSECs will damage hepatic microcirculation and increase hepatic vascular resistance, which will lead to portal hypertension and accelerate the development of MASLD (61). Recent studies showed that LSECs’ fenestration parameters—frequency, porosity, and diameter—negatively correlated with circulating FFAs levels. The progression from simple steatosis to steatohepatitis involves white blood cells adhering to the walls of sinusoidal blood vessels, then storming into the liver tissue to trigger the inflammatory response (62, 63).

In the early stages of MASLD, LSECs may retain anti-inflammatory properties. For example, LSEC-derived nitric oxide suppresses KCs activation in mice subjected to a short-term (8-week) HFD. Similarly, pro-inflammatory chemokines involved in monocyte and macrophage migration via MAPK-dependent pathways are downregulated in human and mouse LSECs exposed to FFA for a brief period (16 hours) (64). Human LSECs generally have low levels of the intercellular adhesion molecule-1 (ICAM-1), but stimulation with IFN-γ and TNF-α upregulates, boosting their production of MHC class II, CD40, and vascular cell adhesion molecule-1 (VCAM-1). Despite this homeostatic role, LSECs can promote inflammation in chronic liver disease. Following heparin-induced fibrotic injury in mice, antigen presentation by LSECs triggers a cascade that increases IFN-γ, IL-6, and TNF-α production and drives T-cells toward a more immunogenic phenotype (65). This shift in inflammatory mediator secretion recruits immune cells and establishes a microenvironment conducive to liver injury. Positioned to receive blood from both the intestinal and systemic circulations, LSECs are uniquely equipped to clear and metabolize blood-borne proteins and lipids. In cooperation with KCs, these cells form the body’s primary clearance system and generate enduring APCs, exhibiting scavenger functions while presenting antigens through MHC class II and I to activate CD4+ or CD8+ T cells (66). Animal studies show LSECs, which normally express low MHC class II, can activate naive CD4+ T cells physiologically, inducing interferon-γ(IFN-γ), interleukin-4(IL-4), and IL-10 production. However, LSECs cannot drive these T cells to differentiate into Th1 effectors (67). In rodent MASH models, LSECs exhibit a pro-inflammatory state, increasing adhesion molecules like ICAM-1 and VCAM-1, and releasing inflammatory cytokines like TNFα, IL-6, IL-1, and MCP-1 as the disease progresses. LSEC-derived inflammatory factors activate neighboring KCs and promote inflammation by facilitating leukocyte recruitment, adhesion, and migration. *In vitro* studies confirm that stimulation of LSECs with ox-LDL and FFAs (palmitate) activates NF-κB and TLRs pathways, respectively (63). MASH-associated inflammation involves mediators such as MCP-1, IL-1β, IL-6, TNF-α, and adhesion molecules, including VCAM-1, ICAM-1, and vascular adhesion protein-1 (VAP-1), contributing to systemic dysregulation. Inflammatory signals enhance the recruitment and activation of white blood cells, such as neutrophils and macrophages, in the liver, which in turn intensifies local inflammation and damage. Furthermore, by failing to maintain HSCs’ quiescence, dysfunctional LSECs produce fibrogenic mediators such as hedgehog signaling molecules that promote liver fibrosis (68). Beyond their roles in microcirculation and immune cell recruitment, recent spatial transcriptomic analyses have uncovered an additional dimension of LSEC involvement in MASLD fibrosis. By unbiased deconvolution of spatial transcriptomic data from the same 61 human livers, Li and colleagues identified a fibrosis-associated gene program that is specifically enriched in advanced. Notably, this gene program suggests profibrotic crosstalk between central vein endothelial cells and HSCs within fibrotic regions, indicating that fibrosis is not uniformly driven throughout the liver but rather emerges in spatially restricted microenvironments. This finding reveals that LSECs, particularly those located around the central vein, may actively participate in fibrogenesis through paracrine signaling to adjacent HSCs, highlighting the central vein as a critical niche for fibrotic progression. Beyond endocytosis and antigen presentation, LSECs also play a critical role in mediating leukocyte adhesion and tissue infiltration. Chemokines and adhesion molecules such as VAP-1 for monocytic and T cell recruitment, CCL2 for monocytes, CXCL10 for T cell recruitment, and CXCL16 for NKT cell trafficking, underpin this process (69).

#### 2.2.2 Dendritic cells

Dendritic cells (DCs) are bone marrow-derived cells present in blood, epithelial, and lymphoid tissues. Distributed throughout the body, they function as sentinel cells against infection and are crucial for maintaining homeostasis. DCs, key APCs, migrate through hepatic sinusoids and traffic to lymphoid tissues such as lymph nodes (70). They can be classified into immature, mature, and regulatory populations based on their functional status and maturation level. Regulatory dendritic cells (DCregs) have been extensively investigated in preclinical models and early-phase human clinical trials, particularly in transplantation and autoimmune diseases. Conventional DCs develop from bone marrow hematopoietic stem cells through intermediate stages, including pro-cDCs and dendritic cell progenitor (CDP). This developmental process requires Flt3L–Flt3 signaling. These precursors exit the bone marrow, reside in lymphoid and non-lymphoid tissues, and subsequently differentiate into either conventional type 1 dendritic cell (cDC1) or type 2 dendritic cell (cDC2).cDC1 is defined by the expression of the C-type lectin receptor Clec9a and the chemokine receptor XCR1, whereas SIRPA is a conserved marker of cDC2 across species (71). In mice, cDC1 predominantly comprises CD8α^+^ or CD103^+^ DCs, whereas in humans, the equivalent population is CD141^+^ (BDCA-3^+^) DCs. These cells are highly proficient in cross-presentation, activating CD8^+^ T cells and promoting Th1-polarized immune responses through the secretion of IL-12, thereby playing essential roles in antiviral and antitumor immunity. By contrast, cDC2 in mice mainly consists of CD11b^+^ DCs, with the human counterpart being CD1c^+^ (BDCA-1^+^) DCs. cDC2 preferentially stimulates CD4^+^ T cells, induces Th2 and Th17 responses, and contributes to host defense against bacterial and parasitic infections. cDC1 is enriched in peripheral tissues, including the skin, intestine, lungs, and liver, whereas cDC2 is broadly distributed in the blood and secondary lymphoid organs (72, 73).

Human DCs are typically isolated from peripheral blood mononuclear cells, differentiated by GM-CSF and IL-4, and mature into dendritic cells following antigen uptake and maturation. Human DC subsets can be identified by the expression of CD11c, CD123, and IL-3Rα, and they also carry CD45 and HLA-DR. Myeloid DCs are crucial antigen-presenting cells. They have high expression of CD11c and low expression of CD123. plasmacytoid dendritic cells (pDCs) express Toll-like receptors 7 and 9, as well as CD11c and CD123. In human HCC liver tissues, the balance and function of CD141+ dendritic cells are disrupted; these cells normally express CLEC9A, ILT3, and ILT4 in healthy liver (74). However, when they encounter an inflammatory environment, DCs will mature, enhance the ability to recruit monocytes, trigger the production of inflammatory cytokines and chemokines, and perform functions including responding to Toll-like receptors, activating NKT cells, and promoting T cell proliferation; Mature DCs also secrete inflammatory mediators, such as TNF-α and IL-6, which can activate stellate cells. Compared with non-obese or slightly obese patients, increased infiltration of CD11c+ DCs is observed in the liver tissue of MASLD patients. In mouse models of MASH, Liver DCs contribute to MASH pathogenesis by accumulating early disease stages and secreting both proinflammatory cytokines and the anti-inflammatory cytokine IL-10, highlighting their dual role in inflammation regulation. These cells induce local inflammation through Toll-like receptors and a pattern recognition mechanism that can recognize pathogen-associated molecular patterns. In mouse MASH models, both CD103+ cDC1 and CD11b+ cDC2 are present in the liver and increase in number with steatohepatitis, but their specific roles in disease development remain unclear (75). In human MASLD, increased cDC1s levels in the liver are associated with greater disease severity. By contrast, hepatic triglyceride levels in Batf3−/− mice lacking cDC1s increased after MASLD induction, but liver damage was similar to that in the control group (76). Paired peripheral blood and liver fine-needle aspiration samples from a comprehensive human MASLD/MASH cohort have enabled the construction of a unique single-cell RNA sequencing atlas spanning the full disease spectrum. Recent single-cell transcriptomic studies have substantially advanced the understanding of dendritic-cell heterogeneity in MASLD. Martin et al. identified a distinct population of S100^hi^HLA^lo^type2 conventional dendritic cells enriched in MASH livers, accompanied by enhanced immunoregulatory programs involving monocytic myeloid-derived suppressor cells and TREM2^+^S100A9^+^ macrophages (77). Spatial transcriptomic analyses further demonstrated stage-specific spatial redistribution of dendritic cells during MASLD progression in resected or biopsied liver samples obtained from 61 patients. The proximity of dendritic cells to T-cell-enriched regions within fibrotic niches was closely associated with local immune activation states (24).

## 3 Adaptive immune cells

The adaptive immune system is an evolutionarily conserved defense mechanism that plays an essential part in maintaining bodily homeostasis and contributing to disease pathogenesis in different tissues. In MASLD, T cells and B cells play a critical role in the progression of liver inflammation. Type 17 T helper (Th17) cells are important contributors. They proliferate in the liver, produce IL-17, and differentiate into pathogenic subsets that promote steatohepatitis and fibrosis. The equilibrium between pro-inflammatory Th17 cells and regulatory T cells (Tregs) is disrupted, resulting in a proinflammatory microenvironment. B cells exert dual functions. Conventional B2 cells infiltrate the liver, secrete antibodies, activate T cells, and produce pro-inflammatory cytokines such as TNF-α and IL-6, thereby exacerbating tissue injury. Alternatively, regulatory B cells (Bregs) predominantly exert protective effects by the secretion of the anti-inflammatory cytokine IL-10. The complex interaction between these proinflammatory and regulatory immune populations forms a complex immunological network, which sustains liver inflammation persistence and contributes to the progression of severe liver fibrosis and HCC.

### 3.1 T cells

CD4-positive T helper cells regulate the immune system by balancing pro- and anti-inflammatory responses throughout the body. The CD4 protein functions as a TCR co-receptor, facilitating antigen recognition via the presentation of MHC class II molecules. These diverse subsets can alter their phenotype upon appropriate stimulation, with considerable functional variation existing across T helper subsets. Th1 cells, which are pro-inflammatory and produce IL-2, TNF-α, and IFN-γ, express the transcription factor T-bet. IL-12 and IFN-γ promote their differentiation via STAT4 and STAT1 signaling activation. In HFD-induced obesity models, Th1 cell presence increases in subcutaneous and visceral fat compared to mice on a standard diet. Inhibiting Th1 function in IFN-γ and T-bet mutant mice reduces adipose tissue inflammation and improves glucose tolerance (78). Th2 cells facilitate wound healing and mediate defense against parasite and helminth infections. The cytokines IL-4 and IL-2, acting through STAT6, are essential for Th2 differentiation. Th2 cells appear to exert anti-inflammatory effects in obesity-associated disorders. Winer et al. observed fewer Th2 cells in mice fed an HFD compared to other T helper subsets. They further demonstrated that lymphodeficient HFD-fed animals receiving CD4+ T lymphocytes from healthy donor mice exhibited lower serum lipase levels, reduced insulin resistance, and decreased total body weight (79). Th22 cells uniquely generate IL-22 independently of IL-17 and other key cytokines. Their production of IL-22 is stimulated by aryl hydrocarbon receptor (AHR) activation. Th22 development is enhanced by IL-6 and TNF-α but suppressed by TGF-β. Obese and type 2 diabetic subjects exhibit increased Th22 cells and elevated IL-22 levels in adipose and blood tissues compared to healthy controls. Proinflammatory Th17 cells express the distinctive transcription factors STAT3 and retinoic acid receptor-related orphan receptor gamma-t (RORγt). They mainly secrete some cytokines called IL-17A, IL-17F, IL-22, and IL-23, which can cause inflammation. By binding to widely distributed receptors, IL-17 cytokines can promote epithelial cells, endothelial cells, and monocytes to release inflammatory mediators, thus amplifying the inflammatory response. Th17 differentiation is driven by cytokines including TGF-β, IL-6, IL-21, and IL-23, which activate STAT3, while IL-1β and TNF-α play an auxiliary role in this process.

In human and mouse studies, different Th17 subgroups exhibit unique gene expression and metabolic characteristics. These cells can express CXCR3 and produce cytokines such as IL-17A, IFN-γ, and CCL5, but they produce less IL-10, which makes them different from the conventional Th17 cell. In the pathological process of MASLD, the change of the intracellular metabolic pathway is very important to regulate the inflammatory capacity and IL-17 response of Th17 cells. In murine MASH livers, Th17 cells constitute the predominant immune cell population. In mouse MASH models, selective knockout of HIVEP1 in IL−17A+ CD4+ T cells impairs Th17 differentiation and thereby slows MASH progression. HIVEP1 mainly controls the differentiation of TH17 cells and the synthesis of cytokines. HIVEP1 is a key regulator of ornithine decarboxylase 1(ODC1), a key enzyme in polyamine metabolism (80). Th17 cells can further differentiate into inflammatory liver CXCR3+Th17 cells (ihTh17), which contribute to disease progression of MASLD. Increased expression of hepatic Th17 cells and IL−17 has been observed in both mouse MASH models and human MASH patients. In mouse models of MASH, DNA damage in hepatocytes triggers Th17/IL−17A−mediated inflammation and promotes fatty acid release. These fatty acids are then stored in the form of liver triglycerides, which promotes the development of MASH (81). In mouse MASH models, the number of Th17 cells increases during the early stage of MASH and during fibrosis progression, whereas Th22 cell levels peak between the first and second waves of Th17 cell expansion. The change of Th17/Th22 cell population is accompanied by the increase of the production of IL-6, TNF-α, TGF-β, and CCL20. IL-17(-/-) mice are protected from MASH development, but there are many Th22 cells in their liver (82). In the liver of MASH mice, CD4+ T helper cells (especially TH1 and TH17 subsets) will increase. Although the elimination of CD8+ T cells will accelerate MASH-related HCC, the elimination of CD4+ T cells will promote tumor growth and reduce the efficacy of immunotherapy (83). When obese, visceral adipose tissue will produce more pro-inflammatory cytokines, such as IL-6, in the presence of IL-17A (84).

#### 3.1.1 Treg cells

Regulatory T (Treg) cells are a subset characterized by Foxp3 expression, which is essential for maintaining immune homeostasis. Treg cells primarily suppress immune responses by secreting IL-10, consuming IL-2, and inhibiting the maturation and function of APCs (85). In healthy adult mice, Treg cells account for 4-8% of CD4+ T cells in the liver (86). Treg cells in the liver can also alleviate liver fibrosis caused by chronic inflammation. For example, CCl4-induced chronic liver injury increases the proportion of Treg cells in the liver, but not in lymphoid tissue (87). The balance between Th17 and Treg cells plays a key role in the pathogenesis of MASLD by regulating immune response and metabolic processes. TGF-β, in the absence of IL-6, promotes Treg cell expansion, enhanced by IL-2 and retinoic acid via STAT5 activation. While low TGF-β concentrations synergize with IL-6 to drive Th17 differentiation, high TGFβ concentrations induce Treg cell differentiation. During hepatic fibrosis, elevated levels of IL-6 and TGF-β activate HSCs to produce ECM proteins, which promote Th17 cell expansion and disrupt the Th17/Treg balance (88). In adult MASLD patients, Treg cell numbers are decreased, and Th17 cell numbers are increased within the portal vein or portal tracts, whereas pediatric MASLD patients show elevated Treg counts. A Th17/Treg imbalance is also evident in cirrhosis. The peripheral blood of compensated cirrhosis patients exhibits a reduced Treg frequency and an increased Th17 frequency, resulting in a lower Treg/Th17 ratio that may serve as a potential diagnostic biomarker. Patients with non-cirrhotic MASLD have fewer hepatic Treg cells than healthy controls, with a more pronounced reduction observed in those with MASH. Consequently, the hepatic Th17/Treg ratio helps distinguish MASH patients from those with simple steatosis (89).

The specific role of Tregs in MASH development remains controversial. The pathogenic versus protective role of Tregs depends on disease stage, anatomical location, cellular plasticity, and the balance between their suppressive and tissue-repair functions. The outcome is a continuum, not a binary. One study investigated the cellular mechanisms driving increased Treg frequency across multiple MASH models. Researchers employed various mouse models of MASH-induced HCC, including a choline-deficient HFD combined with diethylnitrosamine injection and diet-induced models. They observed a decline in overall CD4+ T cell frequency alongside a rise in the proportion of Tregs (CD4+Foxp3+), indicating that an immunosuppressive microenvironment forms prior to overt disease development (90). Treg-specific deletion of amphiregulin (Areg) protected mice from MASH-induced glucose intolerance, a process dependent on EGFR signaling to HSCs. These findings suggest that Treg-driven tissue repair can trigger detrimental adaptive responses in chronic liver disease (91). Furthermore, feeding BALB/c mice a high-fat, high-carbohydrate diet increased hepatic Treg frequency. Conversely, adoptive transfer of Tregs exacerbated experimental MASH. The tumor-promoting role of Tregs on MASH progression is unclear, though they may support tumor growth by suppressing immune surveillance. A study demonstrated that the Nr4a family modulates Treg proliferation and function in the liver during MASH progression (92). MASLD enhances regulatory T cell adhesion to tumor cells via CD29 upregulation, promoting epithelial-mesenchymal transition and progression to hepatocellular carcinoma. Given their immunosuppressive properties, Tregs may facilitate immune escape and have been reported to undergo clonal expansion in HCC (93). The application of single-cell technologies has substantially deepened the understanding of hepatic Treg-cell biology in MASLD (77). Martin et al. reported that enrichment of hepatic regulatory T cells represents a characteristic immunoregulatory program accompanying MASH progression (77). Integrated analyses combining single-cell RNA transcriptomics with T-cell receptor sequencing further demonstrated that Treg cells with enhanced TIGIT and IL-10 expression undergo clonal expansion during MASH progression, while the Nr4a family of orphan nuclear receptors has been identified as a key regulator of intrahepatic Treg proliferation and function.

### 3.2 B cells

#### 3.2.1 B2 cells

B lymphocytes develop in bone marrow and migrate to secondary lymphoid organs such as lymph nodes and spleen. Their precursor cells generate a diverse repertoire of B cell receptors (BCRs) through V(D)J recombination of immunoglobulin heavy and light chains. After activation, these cells differentiate into memory B cells or plasma cells. B2 cells promote inflammation through cytokine secretion, including IL-6, IL-8, IFN-γ, and TNF-α, which establish inflammatory conditions (94). B2 cells produce immunoglobulin G (IgG), which contributes to the activation of T cells and macrophages. The presence of B cells accumulating in adipose tissue may contribute to the development and propagation of insulin resistance, which tends to emerge and spread, both in the immediate vicinity and throughout the entire body (95). In the liver, resident B cells are predominantly IgM+IgD+ B2 cells and are a source of IL-6 and TNF-α (96).

In mice, dietary methionine and choline deficiency—a regimen that induces liver inflammation but also causes substantial weight loss—alleviates hepatitis and fibrosis in MASLD upon B2 cell depletion. A diet high in high-fat high-choline (HFHC) content increases B2 cell prevalence and decreases B1b cell ratio, contrasting with a standard choline diet (97). In the MASH model, B2 cells infiltrate the liver. These cells secrete elevated levels of TNF-α and IL-6, thereby promoting hepatic inflammation and exhibiting pro-inflammatory effects. In the MASH model, if B2 cells are removed, the activation of Th1 cells can be reduced, and the level of IFN-γ in the liver can be reduced, thereby attenuating inflammation (98). In MASH patients, plasma cells upregulate MHC class II molecules preceding T cell infiltration in the liver, a process that coincides with B-cell activation. Interference with B2 cells further reduces TH1 cell activation and IFN-γ production by CD4+ T cells. Genetically engineered mice lacking B2 cells and overexpressing a soluble TACI-Ig receptor exhibited markedly impaired plasma cell development and failed to induce Th1-mediated activation of hepatic CD4+ T cells. These TACI-Ig mice developed milder steatohepatitis and delayed fibrogenesis. Correspondingly, BAFF inhibition with the Sandy-2 antibody suppressed hepatic B2 cells and notably improved MASH (99). Single-cell RNA sequencing reveals that B2 cells are substantially more abundant in fibrotic livers. Hepatic fibrosis activates factors such as Fos, Myc, Jun, and inflammatory indicators active, and then start some natural gene and biological pathways, such as B cell activation, proliferation, MAPK signaling, NF-κB, and Toll-like receptor pathways (100)Spatial multi-omics analyses of human MASLD liver tissues further demonstrated that B-cell accumulation is spatially organized, with IgG^+^ plasma cells enriched within fibrotic and periportal regions, where they co-localize with activated fibroblasts and T-cell aggregates (24).

#### 3.2.2 Bregs

Bregs are a phenotypically and functionally heterogeneous subset of B cells. No single marker can definitively identify all Bregs; instead, they are characterized by a combination of features, such as the expression of CD19, CD25, and CD1d, though these markers are not exclusive to Bregs. Some Breg populations may also express CD24. Bregs play an important role in maintaining immune homeostasis, which can help maintain immune balance. They release anti-inflammatory mediators such as IL-10, IL-35, IDO, and Granzyme B (101). In transplantation models, Bregs have been shown to improve glucose metabolism and attenuate inflammatory reactions in adipose tissue, leveraging their inherent anti-inflammatory capacity (102). Conversely, Bregs can suppress antitumor immunity via IL-10 production and directly promote hepatocellular carcinoma growth through interactions with tumor cells. A HFD reduces the number of Breg cells and IL-10 in the liver of wild-type mice, but it increases them in IgMi mice, especially the number of cells such as CD5+ IL-10-high increased by four times. In human samples with severe liver fibrosis, studies have shown that IL-10 positive cells decreased, but IgG and stromal B cells show increased accumulation (103). The IL-10 production by Bregs may be linked to the unique hepatic phenotype observed in IgMi mice. Flow cytometry confirmed that an HFD reduced Breg frequency in wild-type mice but increased it in IgMi mice, specifically amplifying the IL-10-producing subset (104). Bregs are not uniformly protective. Their function depends on phenotypic definition, IL−10 production capacity, microenvironmental stress, and the balance with conventional B2 cells. Therapeutic Breg expansion must consider their functional plasticity.

TPH cells recruit B cells via CXCL13 secretion and modulate their differentiation through TGF-β−induced IL−10 production, fostering an immunosuppressive microenvironment that promotes hepatocellular carcinoma progression (105). Karl et al. observed that although MASLD results in the loss of IL-10-secreting Bregs, HFD feeding elevates their frequency in IgMi mice and slows disease progression. These findings indicate that the pathogenic B cells exacerbate MASLD progression by producing IgG2c antibody, while the ability of Bregs to secrete IL-10 has a protective effect (106). In mice with B cell deficiency, it was found that the increased IL-10 in the liver came from the Bregs (107).

### 3.3 CD8^+^ T cells

CD8^+^ T cells are central effectors linking chronic hepatic inflammation to MASLD progression. Under metabolic stress and persistent antigen exposure, they accumulate in the liver, promoting hepatocyte injury via perforin/granzyme cytotoxicity and pro-inflammatory cytokines, with the steatotic microenvironment sustaining activation and aggravating fibrosis (108).Chronic antigen exposure induces exhaustion, marked by PD-1, TIM-3, and LAG-3 upregulation, impaired effector function, and metabolic reprogramming, reducing immune surveillance against MASLD-associated HCC (109).Martin et al. generated a single-cell atlas spanning the full MASLD-MASH spectrum in human liver and peripheral blood and demonstrated that hepatic CD8^+^ T-cell activity increased with inflammation but declined during fibrosis progression, accompanied by acquisition of an exhausted phenotype with upregulated PD-1, TIM-3, and TIGIT expression (77).Pfister et al. further reported the accumulation of unconventionally activated exhausted CD8^+^PD1^+^ T cells in MASH-associated HCC models, leading to impaired tumor immune surveillance; moreover, patients with MASH-related HCC exhibited poorer overall survival following immune check point inhibitor therapy compared with patients with HCC of other etiologies (108)MASLD-to-HCC progression is linked to reduced intrahepatic CD8^+^ T cells but expansion of PD-1^+^/CXCR6^+^ subsets interacting with Kupffer cells and neutrophils (110). CD8^+^ tissue-resident memory T (Trm) cells also exert direct regulatory effects during disease progression. During MASH resolution, the liver becomes enriched with CD69^+^CD103^-^ CD8^+^ Trm cells maintained by IL-15. These cells recruit hepatic stellate cells in a CCR5-dependent manner and sensitize them to FasL-Fas-mediated apoptosis. In patients with MASH, increased abundance of CD69^+^ CD8^+^ Trm cells within fibrotic regions correlates with fibrosis regression (111).

CD8^+^ tissue-resident memory T (Trm) cells regulate fibrosis resolution. In MASH resolution, IL-15–maintained CD69^+^CD103^-^ Trm cells recruit HSCs via CCR5, sensitizing them to FasL-Fas apoptosis, correlating with fibrosis regression in patients (111). METTL3 suppresses antitumor CD8^+^ T-cell activity through m^6^A-modified SCAP translation, enhancing cholesterol biosynthesis; inhibition of METTL3 combined with anti-PD-1 restores cytotoxicity in human, murine, and *in vitro* models (112). Spatially heterogeneous immune-cell distribution has also been described in MASH-associated HCC. T cells, myeloid-derived suppressor cells (MDSCs), and tumor-associated macrophages (TAMs) are enriched in adjacent non-tumorous tissues but progressively decrease toward tumor regions. Cell-cell interaction analyses revealed specific connections between exhausted PD-1^+^ CD8^+^ T cells and PD-L1^+^/ICOS^+^ MDSCs or TAMs, whereas such interactions were absent in viral HCC. Tumor cells simultaneously exhibited relatively low PD-L1 expression (113). In addition, METTL3 suppresses antitumor immunity by reducing infiltration of GZMB^+^ and IFN-γ^+^ CD8^+^ T cells in human samples, *in vitro* systems, and murine models. Mechanistically, METTL3-mediated m^6^A modification of SCAP mRNA enhances its translation, activates cholesterol biosynthesis, and promotes secretion of cholesterol and cholesterol esters, thereby impairing CD8^+^ T-cell function. Targeting METTL3 in combination with anti-PD-1 therapy synergistically restores cytotoxic CD8^+^ T-cell activity and induces tumor regression (112). Spatial analyses show T cells, MDSCs, and TAMs enriched in non-tumorous tissue but decrease toward tumors, with exhausted PD-1^+^ CD8^+^ T cells interacting with PD-L1^+^/ICOS^+^ MDSCs or TAMs in MASH-HCC (113).Transcriptomic/secretomic profiling of 409 MASLD patients identified PLS-MASLD signatures predicting HCC, associated with portal IDO1^+^ DCs and dysfunctional CD8^+^ T cells, modifiable by bariatric surgery, statins, and IDO1 inhibitors (114).In FFC-induced murine models, RAGE inhibition reduced macrophage-mediated CD8^+^ T-cell activation and liver injury (115). Anti-CD122 antibodies or exercise reduced pathogenic CD44^+^CXCR6^+^PD-1^+^ CD8^+^ T cells, restored function, and suppressed tumor progression in murine MASH-HCC models (115). Type I IFN signaling promotes IFN-γ and TNF-α production by CD8^+^ T cells and exacerbates hepatic inflammation, while STING deficiency suppresses activation and attenuates disease progression. CD8^+^ T cells cooperate with NKT cells via LIGHT-LTβR/NF-κB to drive MASH-to-HCC transition, with increased infiltration in MASH livers (116). Elevated hepatic PD-L1 expression has also been reported in MASLD and is induced by FFAs through the ROS/ZNF24 pathway, while PD-L1 upregulation may limit FFA-induced hepatocyte injury (117). Collectively, CD8^+^ T cells undergo complex dynamic alterations and metabolic-immune crosstalk throughout MASLD/MASH and HCC progression, highlighting their potential as important therapeutic targets.

## 4 Multiple interconnected crosstalk between innate and adaptive immunity in MASLD

In MASLD, the crosstalk between innate and adaptive immunity is orchestrated through multiple interconnected pathways. Receptor-ligand interactions such as CD28/B7 and OX40/OX40L are crucial for T cell activation and function within the liver. Cytokine networks represent another key mechanism of intercellular communication (**Table 1**). A dysregulated type I interferon(IFN) response promotes pathogenic CD8^+^ T cell accumulation, whereas IL-11 directly activates HSCs to drive fibrosis, serving as a key mediator linking innate and adaptive immunity that amplifies inflammation by activating macrophages and neutrophils while also influencing Th17 cell differentiation. Furthermore, the gut-liver axis facilitates a continuous interaction in which gut-derived microbial products activate hepatic innate immune cells via pattern recognition receptors, subsequently allowing bacterial metabolites to modify adaptive T and B cell responses. These bidirectional interactions collectively contribute to MASLD progression. In addition to the classical innate and adaptive subsets discussed above, several bridging populations at the innate–adaptive interface—including CD8^+^ T cells, NK cells, MAIT cells, γδ T cells, and myeloid-derived suppressor cells—play critical roles in MASLD pathogenesis but are not covered in detail due to space limitations. Their key features, disease-associated functions, and major crosstalk mechanisms are summarized (**Table 2**).

**Table 1:** Effects of dysregulated immune crosstalk pathways in MASLD mouse models.

Pathway	Model used	Effects on MASLD	Reference
CD28	CD28^-/-^ mice (C57BL/6)	Reduce hepatic steatosis, hepatitis, and hepatocellular damage.	(233)
B7.1 and B7.2	B7.1^-/-^B7.2^-/-^mice (C57BL/6)	Exacerbate hepatic steatosis; Increase hepatic inflammation; Alleviate hepatocyte damage.	(119)
4-1BB/4-1BBL	4-1BB^-/-^mice(C57BL/6)	Reduce hepatic steatosis; Alleviate liver inflammation.	(121)
OX40/OX40L	OX-40^-/-^ mice(C57BL/6)	Reduce hepatic steatosis; Alleviate liver inflammation	(125)
CD40/CD40L	CD40^fl/fl^ CD11c-Cre mice(C57BL/6)	Increase hepatic steatosis; Exacerbate hepatocyte damage; Reduce the number of liver-derived regulatory T cells.	(129)
Fas/FasL	Fas^fl/fl^ albumin-Cre mice (C57BL/6)	Improve hepatic steatosis.	(234)
IFN-I/IFNAR	IRF5t^fl/fl^ Lyz2-Cre mice(C57BL/6)	Improve hepatocyte damage and hepatic inflammation, liver cirrhosis.	(138)
APRIL	APRIL^-/-^mice(C57BL/6)	Reduce hepatocyte damage.	(235)
BAFF/BAFF-R	BAFF mice (C57BL/6)	Improve hepatocyte damage and hepatic inflammation, liver cirrhosis.	(236)

**Table 2:** Bridging immune cell populations at the innate–adaptive immune interface in MASLD/MASH.

Cell population	Immunological localization and phenotypic features	Defined roles in MASLD/MASH	Key crosstalk mechanisms	References
CD8^+^ T cells	Tissue-resident memory cells (Trm); high expression of CXCR6 and PD-1	Early stage: antigen-independent “self-reactivity,” inducing hepatocyte apoptosis via direct cytotoxicity; late-stage fibrosis: reduced cytotoxicity and marked immune exhaustion	Metabolites (e.g., acetate) and IL-15 alter their transcriptional network, triggering MHC-I–independent killing; hepatocyte death releases DAMPs, activating Kupffer cells and HSCs	(109, 111)
NK cells	Highly abundant innate lymphocytes in the liver microenvironment	Early fibrosis: clear senescent or activated HSCs, exerting antifibrotic protection; during MASH progression: NK cell–driven cytotoxicity is enhanced	Recognize and eliminate activated HSCs via NKG2D receptor; macrophages/DCs secrete IL-15 and other proinflammatory factors to remodel and amplify NK cell cytotoxic networks	(77)
MAIT cells	Mucosal-associated invariant T cells, dependent on MR1 to recognize microbial metabolites; markedly accumulate in the liver during MASLD	Early lesions: secrete large amounts of IL-17A, displaying proinflammatory and profibrotic phenotypes; cytotoxicity is significantly enhanced, exacerbating hepatic parenchymal inflammation and damage	Early IL-17A signaling acts on HSC surface receptors to promote proliferation and collagen gene expression; MASLD-related PUFAs impair MAIT cell mitochondrial respiration, inducing cellular exhaustion	(237)
γδ T cells	Located at the innate–adaptive immune interface; mainly IL-17–producing γδT17 subset in the liver	High-fat/high-sugar metabolic stimuli induce rapid intrahepatic expansion; major source of IL-17A in early MASH, directly driving steatohepatitis and systemic metabolic dysregulation	Secrete IL-17A to stimulate hepatocytes to release CXCL1/CXCL2, recruiting and activating neutrophils; gut-derived LPS activates macrophages via TLR4 to release IL-1β/IL-23, maintaining sustained γδT17 activation	(147, 238)
MDSC cells	Immature myeloid cells abnormally expanded in metabolic inflammation and lipotoxic microenvironments; monocytic M-MDSCs are enriched in MASH	Establish heightened immunoregulatory programs, hindering host protective immune responses; promote HSC activation and ECM deposition via TGF-β secretion	Engage in close crosstalk with hepatic Tregs to form an immunosuppressive network; high ARG1 expression depletes nutrients such as arginine in the microenvironment, inhibiting effector T cell activation and proliferation	(239)

### 4.1 Receptor/ligand-driven communication pathways

#### 4.1.1 CD28/B7.1 and B7.2 signaling pathways

The B7 molecule of the CD28 family is very important for full activation and clonal proliferation of T cells. These molecules also deliver inhibitory signals to help maintain immune balance, modulate inflammatory responses and immune homeostasis, and promote tolerance. Analysis of TCGA data was used to evaluate the prognostic significance of the B7/CD28 family in patients with HCC. Further analysis revealed that stromal expression of B7-H3 (CD276) was the primary factor associated with poor prognosis in HCC patients. This family mediates dynamic cellular communication where B7H3 expression also significantly influences patient prognosis (118). CD28 deficiency reduces the abundance of pathogenic T cells and Tregs in adipose tissue without affecting macrophage numbers. In mice, CD28 inactivation diminishes T cell activation. In mouse models, combined B7.1/B7.2 deficiency exacerbates insulin resistance and MASH in the context of obesity. Combined B7.1/B7.2 gene knockout worsened obesity-related pathologies by impairing insulin sensitivity in the liver and adipose tissue, compromising glucose tolerance, and accelerating steatohepatitis (119).

#### 4.1.2 BB/4-1BBL signaling pathway

CD137(4-1BB), a glycoprotein of the TNF receptor superfamily, is a membrane-bound protein crucial for immune regulation and is expressed on multiple immune cell types, including NK cells, Tregs, and activated CD4+ and CD8+ T lymphocytes, with a subset of Tregs expressing it constitutively. Several co-stimulatory receptors of T cells, along with their ligand CD137L, are part of the same receptor family. The 4-1BB receptor is essential for generating immunological memory and sustaining effective T cell immune responses. However, 4-1BB agonists can induce severe hepatic inflammation, which may impede clinical progress (120). High-fat diets led to increased weight gain and fat cell proliferation in 4-1BB gene-deficient mice, which also exhibited elevated macrophage and T cell infiltration, alongside higher levels of inflammatory markers such as TNF-α, IL-6, and monocyte chemoattractant protein-1(MCP-1), compared to 4-1BB-positive mice. The mice also showed impaired glucose homeostasis, insulin resistance, and hepatic steatosis (121). Therefore, combining 4-1BB co-stimulation with agonistic antibodies could be a viable therapeutic approach for hepatocellular carcinoma characterized by robust T-cell activation (122).

#### 4.1.3 OX40/OX40L signaling pathway

The OX40 receptor, also known as TNFR OX40 or CD134, functions as a T cell co-stimulator upon activation by its ligand, OX40L (CD134L or CD252). Targeting the OX40-OX40L interaction represents a potential therapeutic strategy for autoimmune disorders. OX40 is expressed on activated T cells and, in mice, on resting regulatory T cells (Tregs). OX40L is present on APCs, activated T cells, and other cell types, including lymphoid tissue-inducing cells, certain endothelial cells, and mast cells (123). In mouse models of liver fibrosis, OX40 expression is significantly elevated in fibrotic liver. Following CCL4 exposure, alanine aminotransferase (ALT) and aspartate aminotransferase (AST) levels rise markedly. This increase in OX40 expression during liver fibrosis correlates strongly with elevated α-smooth muscle actin (α-SMA) levels and collagen fiber deposition. OX40 is likely to impair immune control throughout liver fibrosis development, thereby exacerbating hepatic injury (124). OX40 upregulation in liver CD4+ T cells accompanies MASH development. Inhibition of the OX40L-OX40 axis attenuated liver fibrosis, improved histological disease parameters in MASH mice, and reduced inflammatory markers in human liver tissue models (125). OX40 promotes pro-inflammatory T cell differentiation and sustains liver T cell survival. It aggravates diet-induced hepatic inflammation and may amplify systemic inflammatory responses, contributing to the progression of inflammatory disorders (125).

#### 4.1.4 CD40/CD40L signaling pathway

CD40 ligand (CD40L, also known as CD154) is a key modulator of innate and adaptive immunity. CD40 expression is widely distributed across tissues. While it is typically low or absent in healthy livers and cultured hepatocytes, its expression increases under inflammatory conditions. In MASLD, lipid-laden hepatocytes express CD40. Inflammatory infiltrates and hepatocytes in patients with MASH also express CD40. A genetic deficiency of CD40 or CD154 increases susceptibility to fatty liver, especially under high-fat or lipid-rich dietary conditions. CD154-deficient mice exhibit increased lipid accumulation, while female mice with the CD40 gene knocked out will develop more severe hepatic steatosis (126). Among the bioactive factors released from platelet α-granules is CD40L. This is notable because platelets are the primary source of circulating CD40L, implicated in antitumor immune responses. Research indicates that platelet-derived CD40L exerts antitumor effects in MASLD. Hepatic IL-12 expression was elevated in MASLD mice (127). In MASLD, upregulation of CD40 mRNA and protein expression is associated with reduced severity of hepatic lesions. Liver function markers and lipid profiles improve significantly, accompanied by a significant increase in Treg cell numbers. The regulation of the Th17/Treg cell ratio is also markedly enhanced (128). The CD40-CD40L axis is crucial for regulating immune responses, particularly in obesity-related inflammation. CD40-expressing CD11c+ cells exhibit a dual role: they promote hepatic inflammation during MASH, yet they also induce regulatory T cells that confer protection against metabolic syndrome (129). The paradoxical observations—CD40 deficiency exacerbates steatosis yet CD40 expression correlates with inflammation—reflect cell−type−specific effects. CD40 in hepatocytes may be protective, whereas in CD11c^+^ myeloid cells it drives inflammation. Thus, CD40/CD40L cannot be categorically designated as protective or pathogenic.

#### 4.1.5 Fas/FasL signaling pathway

The Fas/Fas ligand (FasL) system is a key cellular pathway in which FasL activates its receptor Fas to trigger programmed cell death. Early evidence for its role in hepatic homeostasis and disease emerged from studies showing that anti-Fas antibody administration causes rapid mortality, liver failure, and severe hepatocyte apoptosis in mice. Fas/FasL-mediated apoptosis contributes to various pathological processes, including chronic liver disease and hepatocyte dysfunction. This pathway promotes NF-κB p65/PUMA-mediated hepatocyte apoptosis in an autophagy-dependent manner, thereby activating HSCs and driving liver fibrosis (130). In pediatric patients, serum levels of sFas and sFasL are significantly higher in those with MASH than in those without. For predicting biopsy-confirmed MASH, sFasL demonstrated superior diagnostic accuracy. Markers of the extrinsic apoptosis pathway are also elevated in pediatric MASH, suggesting both sFasL and a MASH apoptosis score may serve as novel biomarkers for the condition (131). In both human steatotic livers and mouse models, increased Fas/FasL expression heightens hepatocyte susceptibility to Fas−induced apoptosis, thereby promoting liver damage, cirrhosis, and end-stage liver disease. Conversely, liver-specific Fas deletion in mice alleviates obesity-related hepatic steatosis and insulin resistance (132). Immune cells are the principal source of FasL, among which CD8+ T cells and natural killer (NK) cells serve as the major effector populations mediating Fas/FasL-dependent hepatocyte apoptosis. In the context of CD8+ T cells, activated CD8+ T cells upregulate FasL expression and induce hepatocyte apoptosis through interaction with Fas receptors expressed on the hepatocyte surface. This mechanism represents a direct driver of MASLD/MASH-associated liver injury. In MASH mouse models, acetate generated from fatty acid metabolism promotes the conversion of CD8+ T cells into auto-aggressive T cells expressing CXCR6 and FasL, thereby directly inducing hepatocyte death (133). In addition to CD8+ T cells, NK cells constitute another important source of FasL in MASLD livers. Activated NK cells promote hepatocyte apoptosis and contribute to the progression of steatohepatitis through upregulation of FasL expression. A reported case of NK-cell leukemia demonstrated that malignant NK cells overexpressed functional FasL and were associated with severe liver injury, further supporting the clinical relevance of NK cell-mediated hepatocyte apoptosis through the Fas/FasL axis (134). In MASLD mouse models, CD8+ tissue-resident memory T (CD8+ Trm) cells have also been implicated in the direct resolution of liver fibrosis. Single-cell transcriptomic and FACS analyses revealed an enrichment of CD69+ CD103+ CD8+ Trm cells in the liver during MASH resolution. Reduction of hepatic CD8+ Trm cells maintained by IL-15 markedly delayed fibrosis regression, whereas adoptive transfer of these cells protected mice from fibrosis progression. During fibrosis resolution, CD8+ Trm cells recruited HSCs in a CCR5-dependent manner and rendered activated HSCs susceptible to FasL-Fas-mediated apoptosis. Histological assessment of patients with MASH further demonstrated increased abundance of CD69+ CD8+ Trm cells within fibrotic regions, supporting their functional relevance in humans. Collectively, these findings highlight a previously underappreciated role of hepatic CD8+ Trm cells in fibrosis resolution (111). Another study using MASLD mouse models demonstrated that hepatic NK cells possess intrinsic cytotoxicity against normal hepatocytes, and that the FasL-Fas pathway mediates part of the NK cell-induced hepatocyte killing. FasL expressed on hepatic NK cells binds to Fas receptors on hepatocytes and subsequently induces hepatocyte apoptosis. Moreover, poly I:C treatment enhances the cytotoxic activity of hepatic NK cells, further upregulates FasL expression, and exacerbates hepatocyte death (135).

### 4.2 Cytokine-driven communication pathways

#### 4.2.1 Interferon signaling

IFN signaling pathway, a critical bridge between the innate and adaptive immunity, is central to infection control and immune homeostasis. This signaling cascade is initiated by pattern recognition receptors such as Toll-like receptors and transduced through IFN-α/β receptors, leading to activation of the JAK/STAT pathway. This activation ultimately regulates immune responses by controlling the expression of interferon-stimulated genes (ISGs) (136). In MASLD, an overactive type I IFN response is triggered, as evidenced by elevated IFN-α and subsequent upregulation of Irf7 and ISGs in HFD or methionine–choline-deficient (MCD) diet models. A core pathological function of this dysregulated axis is to disrupt the hepatic immune microenvironment, primarily by driving the accumulation and activation of pathogenic CD8+ T cells. Inhibiting IFN-αR1 or genetic deletion of Ifnar1 in mice markedly improves metabolic parameters and reduces hepatic injury, confirming that the hepatic type I IFN response is a central immune axis driving intrahepatic T-cell pathogenicity and promoting the progression of metabolic syndrome and MASLD (137). Furthermore, activation of the downstream transcription factor IRF5 promotes the pro-inflammatory polarization of hepatic macrophages and drives HSC activation, resulting in extracellular matrix deposition and liver fibrosis. Inhibiting IRF5 exerts a protective effect by inducing anti-inflammatory macrophages, enhancing the release of IL-10 and TGF-β, and expanding regulatory T cell populations. This process reduces hepatocyte apoptosis, thereby preventing MASLD progression to inflammatory and fibrotic stages (138). The effects of type I IFN signaling, however, demonstrate tissue and context specificity.

#### 4.2.2 Interleukin signaling

Interleukin-11 (IL-11) can directly activate HSCs, thereby promoting fibrogenesis. In several preclinical MASH models, the inhibition of IL-11 has shown therapeutic potential because it can improve the core pathological features of fibrosis, inflammation, and steatosis. This inhibition can reduce the release of proinflammatory cytokines and chemokines from activated fibroblasts and myofibroblasts, thus reducing hepatic inflammation and protecting parenchymal cells (139). Anti-IL-11 therapy can also reverse liver fibrosis, change the ratio of tissue inhibitor of metalloproteinases to matrix metalloproteinases (TIMP/MMP), and promote extracellular matrix remodeling. This therapy can also inhibit the expression of genes related to liver inflammation, reduce the migration of immune cells, and reduce the level of TGF-β1 in circulation, supporting its therapeutic potential. Moreover, anti-IL-11 treatment also reduced the triglyceride content in the liver and improved the metabolic parameters of the whole body, such as serum triglyceride, cholesterol, and fasting glucose levels (140). Unlike IL-11, which mainly acts on stromal cells, other interleukins act in MASLD/MASH through different mechanisms (141). On the contrary, IL-1 cytokines are powerful inflammatory messengers in the innate immune system, and play a critical role in starting the inflammatory response in alcoholic and MASLD liver diseases. Mice lacking IL-1α/β show immune protection after fatty liver caused by diet, but their cholesterol levels in the liver and blood may increase, which indicates that there is a complex interaction between lipid metabolism and inflammatory pathways (142). IL-6 exhibits stage-dependent and multifaceted functions during MASLD progression. On the one hand, IL-6 can promote macrophage polarization toward the anti-inflammatory M2 phenotype through activation of the STAT3 signaling pathway. In a chronic hepatitis mouse model, deficiency of IL-6/STAT3 signaling resulted in enhanced macrophage accumulation and aggravated MASH phenotypes, including hepatic steatosis. Moreover, tumor development was strongly associated with massive intrahepatic macrophage infiltration and enhanced lipid deposition in hepatocytes (143). IL-6 contributes to insulin resistance and inflammation in MASLD yet can be hepatoprotective; in HFD-fed mice, anti-IL-6R antibody ameliorated insulin resistance through AMPK-GLUT4 signaling and reversed hepatic steatosis after long-term treatment (144). Under fibrotic conditions, increased IL-6 secretion by Kupffer cells promoted hepatocarcinogenesis, whereas IL-6 depletion enhanced hepatocyte injury and cell death. Studies using DEN/CCl4-treated male mice, IL-6 knockout mice, and wild-type mice further supported these observations. Obesity-induced activation of IL-17 is considered one of the driving factors contributing to the initiation and progression of MASLD, suggesting that IL-17 may represent a potential therapeutic target for liver diseases (145, 146). In MASLD models, Th17- and γδ T cell-derived IL-17 promotes hepatocyte steatosis, HSC-driven fibrosis, and chemokine-mediated recruitment of pro-inflammatory immune cells, with circulating IL-17A^+^ γδ T cell frequency in MAFLD patients correlating positively with liver pathology severity (147). In addition, IL-17 can directly activate hepatic stellate cells and thereby promote fibrosis. In the pathogenesis of MASLD, IL-17 and IL-22 exert opposing functions, with IL-22 primarily exerting protective effects through induction of antioxidant and anti-apoptotic factors (148, 149).IL-10 is an anti-inflammatory cytokine that plays a protective role in MASLD. IL-10 exerts anti-inflammatory and anti-fibrotic effects by suppressing the production of pro-inflammatory cytokines, including TNF-α, IL-1β, and IL-6, reducing myeloid cell recruitment, and inhibiting hepatic stellate cell activation (150).IL-10, produced by Tregs, macrophages, and other immune cells, suppresses inflammation and maintains immune homeostasis. Reduced IL-10 signaling may contribute to persistent hepatic inflammation and MASLD progression. Hu et al. found that advanced MASLD was associated with decreased Treg proportions, an elevated Th17/Treg ratio, and altered plasma IL-10/IL-17A levels, indicating that Th17/Treg imbalance and relative IL-10 deficiency promote disease progression (151).In animal studies, IL-10 inhibited activation of hepatic stellate cells and ameliorated liver injury in MASLD mouse models; however, it did not exert protective effects against insulin resistance (146).

### 4.3 Complement system

#### 4.3.1 C1q/MBL-MAC signaling axis

**Figure 3:**
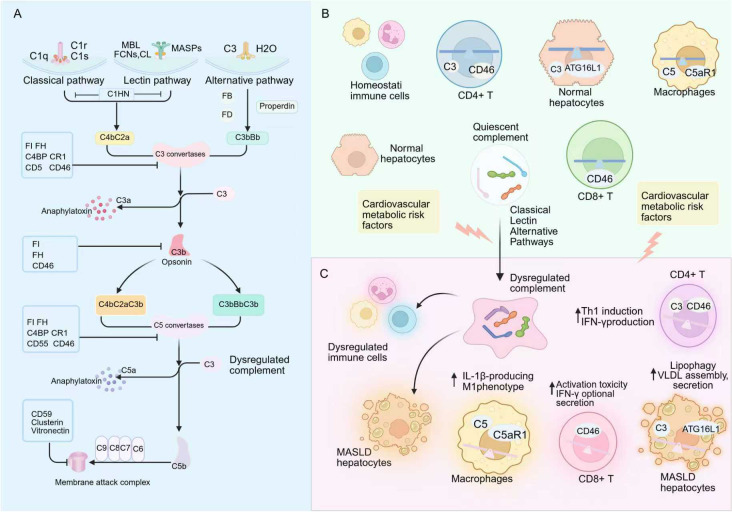
Complement system activation and immune dysregulation in MASLD. **(A)** Activation of complement occurs in three separate ways. The classical methodology requires C1q and C1s; to start, the lectin pathway depends on MBL, FCNs, CL, and MBL-associated serine proteases. The components of the alternative pathway include FB, FD, and properdin. The result of these pathways is the production of C3 invertase-classical/lectin pathway generates C4bC2a, and the alternative pathway generates C3bBB-cleaves C3 into C3a and C3b. Then, C5 invertase (C3bBbC3b) decomposes C5, which helps to assemble MAC. Allergic toxins C3a and C5a will be released in the cascade reaction. A variety of regulatory proteins (F1, FH, C4BP, CR1, CD5, CD46, FI) inhibit invertase activity and prevent MAC formation through multilevel mechanisms. **(B)** Under steady-state conditions, the static complement components and CD46^+^ CD4^+^ T cells will stay next to normal liver cells. **(C)** Cardiovascular metabolic risk factors, like triggers, will activate classical, lectin, and bypass complement pathways, while making CD8^+^ T cells and macrophages abnormal. The maladjusted complement will turn macrophages into M1 type that can produce IL-1β, promote the differentiation of IFN-γ secreting Th1 cells, and directly lead to the damage of MASLD hepatocytes. C3 and CD46 can regulate the effector response of T cells together. CD46^+^ CD4^+^ T cells were activated. CD8^+^ T cells enhanced cytotoxicity and the ability to secrete IFN-γ. Image was partly created with https://biorender.com/. MBL, Mannose-binding lectin; FCNs, Ficolins; CL, Collectins; FB, Complement factor B; FD, Complement factor D;MAC, membrane attack complex; C4bC2a, C4bC2a complex;C3bBb, C3bBb complex; C3,Complement component 3;C3a, Complement component 3a;C3b, Complement component 3b;C5, Complement component 5;C5a, Complement component 5a;FI, Complement factor I;FH, Complement factor H;C4BP, C4b-binding protein;CR1, Complement receptor 1; IFN-γ, Interferon-gamma.

The complement system is a complex network that consists of more than 50 components, plasma proteins, and membrane receptors synthesized mainly in the liver. It is an important part of innate immune defense. It is activated through three different pathways- the lectin pathway, alternative pathway, and classical pathway-to provide key protection against infection (**Figure 3**). This system connects innate immunity and adaptive immunity by coordinating inflammatory response, recruiting leukocytes, and eliminating antigens (152). A Clinical meta-analysis showed that the serum levels of complement C4 and factor B in patients with MASLD were significantly lower than those in healthy controls, with mean differences of 0.04 (95% CI: 0.02–0.07) and 0.22 (95% CI: 0.13–0.31), respectively. In contrast, the level of complement factor D shows no statistically significant difference between groups (153). Complement activation is also evident in liver histopathology. Most patients with MASLD have activated C4d deposition, and about half of them also show C9 deposition related to the membrane attack complex (MAC). These deposits are primarily localized around hepatocytes and are accompanied by macrovesicular steatosis, increased expression of inflammatory cytokines such as IL-6, IL-8, and IL-1β, enhanced hepatocyte death, and neutrophil infiltration. These findings suggest that the extent of complement activation correlates with the severity of hepatic inflammation and disease progression toward MASH (154). A proposed mechanism suggests that excessive complement activation (especially through the alternative pathway) continues to occur throughout the course of MASLD. In the early stage of the disease, it leads to insulin resistance and lipid metabolism disorders, promotes the influx of FFAs into the liver, and causes abnormal triglyceride synthesis and deposition, thereby promoting the development of MASL (155). Excess lipid accumulation induces adipocyte death. These apoptotic cells are recognized by C1q and mannose-binding lectin (MBL), and then further activate the complement system, forming a vicious circle. In addition, the relationship between the terminal components of complement and liver fibrosis is complex; For example, there is a positive correlation between C7 and severe fibrosis in MASH, while the C8γ chain is negatively correlated. As primary targets of complement-mediated injury, hepatocytes are irreversibly damaged through apoptosis and necrosis, which is the key determinant to determine the severity and progress of the disease.

#### 4.3.2 C3/C5a-C5aR1 signaling axis

The complement system maintains the immune homeostasis of the liver, and its active components C3a and C5a bind to the corresponding receptors. C3a/C3aR and C5a/C5aR signaling axes are key mediators of macrophage-mediated inflammation. In the HFD model, C3aR is highly expressed in macrophages of white adipose tissue and KCs of the liver. C3aR deficiency can improve insulin sensitivity, reduce macrophage infiltration, and inhibit M1 polarization, thus alleviating liver injury. Similarly, the C5a/C5aR axis can promote the accumulation and M1 polarization of macrophages in adipose tissue and aggravate insulin resistance (156). Activated macrophages secrete TGF-β, TNF-α, and IL-1β, which promote HSC activation and a profibrotic phenotype and accelerate collagen deposition and liver fibrosis. C5a can directly trigger the activation of the NLRP3 inflammasome, promote the release of IL-1β and TNF-α, and amplify the inflammatory response. In addition to macrophages, these signals can also regulate other immune cells: the C5a/C5aR axis will drive the chemotaxis and respiratory burst of Neutrophils, while C3a has a dual effect on Neutrophils; although the receptor expression is high, it may mediate the anti-inflammatory response (157). The combination of C3a and its receptor C3aR plays a critical role in Th2 cell differentiation, and the activation of C3aR and C5aR together promotes differentiation of Th1 and Th17 cells.C5aR signaling in DCs is particularly critical to the development of Th17 cells (158). C3a/C3aR and C5a/C5aR1 axes promotes pathogenic immune response by regulating the activities of T cells, neutrophils, and macrophages. Beyond its pro−inflammatory and profibrotic roles, complement also exerts homeostatic functions. Net outcome depends on activation intensity, duration, and context. Therapy should aim to restore balance, not achieve complete inhibition.

### 4.4 Gut microbiome

#### 4.4.1 Gut microbiota

**Figure 4:**
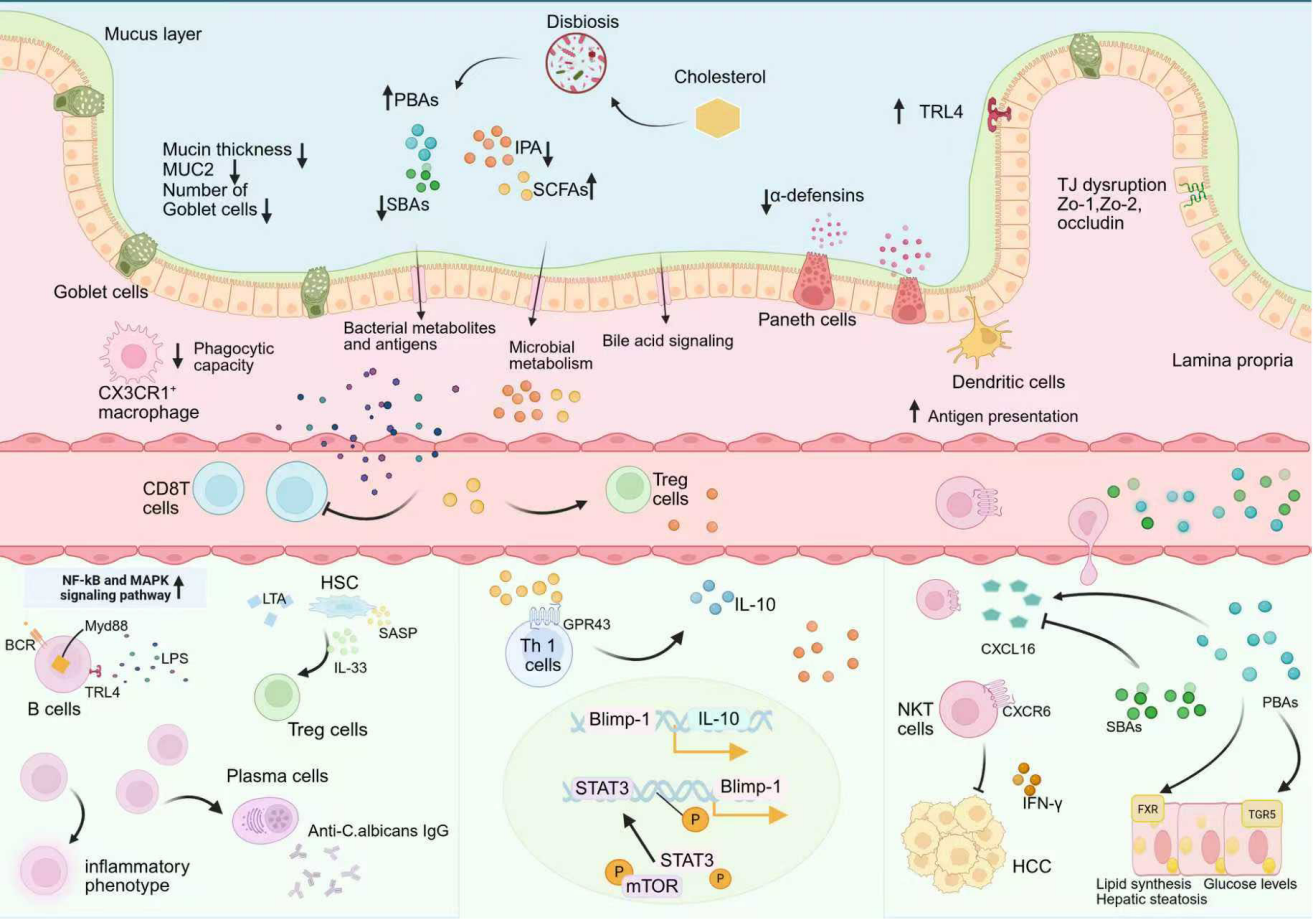
Dynamic changes in gut microbiota during MASLD progression. The manifestations of intestinal barrier damage include the thinning of the mucus layer, the decrease of MUC2, the decrease of goblet cells, and the decrease of the expression of tight junction proteins ZO-1, ZO-2, and occludin. Dysbacteriosis is also accompanied by cholesterol accumulation, α-defensins changes of Paneth cells, and bile acid signal transduction disorder. Bacterial metabolites and antigens will pass through this loosened barrier and be absorbed by CX3CR1^+^ macrophages in the lamina propria. The phagocytosis of these macrophages will change, and the antigen will be presented to CD8^+^ T cells. LTA is able to stimulate the HSCs and induce the SASP and IL-33 release. GPR43 signaling has the ability to enhance Th1 cell phosphorylation and BLIMP-1 up-regulation, and enhance the release of IL-10 and IFN-γ. LPS activates NF-κB and MAPK pathways through TLR4 and MyD88, and promotes B cells to differentiate into plasma cells, which will produce IgG against Candida albicans and show an inflammatory phenotype. NKT cells in the liver will increase CXCR6 and increase IFN-γ through the STAT3 signal. BAs regulate these reactions through FXR and TGR5, which together form the microbial community-liver immune axis, and promote hepatocellular carcinoma related to MASLD. Image was partly created with https://biorender.com/. MUC2, Mucin 2; ZO-1, Zonula occludens-1; ZO-2, Zonula occludens-2; LTA, Lipoteichoic acid; SASP, Senescence-associated secretory phenotype; IL-33, Interleukin-33; GPR43, G protein-coupled receptor 43; BLIMP-1, B lymphocyte-induced maturation protein 1; NF-κB, Nuclear factor kappa-light-chain-enhancer of activated B cells; MAPK, Mitogen-activated protein kinase; STAT3, Signal transducer and activator of transcription 3; TGR5, Takeda G protein-coupled receptor 5; Bas, Bile acids; FXR, Farnesoid X receptor.

The intestine is a critical interface connecting the body with the external environment. It plays a pivotal role in the immune homeostasis and disease development of the liver through the microbial community in the gut-liver axis. The liver receives direct blood flow from the intestine via the portal vein from the intestines via the portal vein, thereby exposing it to microbial byproducts and intestinal metabolites (159). These intestinal microorganisms form a complex network, including viruses, bacteria, fungi, archaea, and protozoa. Alterations in this community regulate the translocation of microbe-associated molecular patterns (MAMPs), thus dynamically affecting the immune response of the liver. For example, in the animal model of liver fibrosis, gut microbiota dysbiosis will reshape the T-cell receptors repertoire and reduce its diversity. The balance between endotoxin and microbial toxins entering the liver will also affect the functional properties of specific T-cell subsets (160). The destruction of intestinal homeostasis will prompt local immune cells to activate NLRP3 inflammasome, leading to endogenous signal molecules such as cardiolipin and cholesterol crystals entering the liver through the enterohepatic circulation (161) (**Figure 4**). Receptors for pattern recognition, like TLRs and NOD-like receptors (NLRs), are the main channels for these transferred microbial by-products to initiate downstream signaling cascades. Following TLR4 recognition of lipopolysaccharide (LPS), proinflammatory cytokine production is strongly induced. Clinical observations reveal elevated circulating endotoxin levels and increased TLR4+ macrophages in the livers of MASH patients. Human genetic studies suggest that susceptibility to MASLD correlates with the expression of genes such as TLR4 and TLR9 (162). By-products of microorganisms, such as short-chain fatty acids (SCFAs), tryptophan derivatives, and bile acids (BAs), as well as pathogen-associated molecular patterns (PAMPs), have a great influence on the immune response. SCFAs can regulate the function of T cells, promote the development of regulatory T cells, or increase the production of IL-22 (163). In patients with MASLD, the balance of BAs is broken, which may make regulatory T cells differentiate more than Th17 cells, thus affecting the inflammatory response. In mice fed a high−fat diet, tryptophan and other metabolites are reduced, thereby diminishing their anti−inflammatory effects on macrophages and hepatocytes (164). The immune activation mechanism also includes that present microbiota-derived lipid antigens in the portal vein through CD1d, thus activating pro-inflammatory γδ T cells to produce IL-17A, which aggravates liver disease (165, 166). In mouse models of MASH, fecal microbiota transplantation alleviates disease, probiotic supplementation delays disease progression, and loss of TLR4 or TLR9 reduces diet−induced hepatic fat accumulation, inflammation, and fibrosis (167). In mouse models, specific probiotics such as *Akkermansia muciniphila* maintain liver health through mechanisms including short−chain fatty acid production and IL−10 release (168). In mice deficient in inflammasome components such as NLRP3 or NLRP6, microbiota dysbiosis directly promotes hepatic fat accumulation and inflammation. This phenotype can be transmitted via microbiota transfer, which clearly confirms the direct influence of intestinal flora on the development of the disease (169). Among the factors translocating from the gut, bacterial components such as lipoteichoic acid (LTA) can stimulate HSCs to induce the senescence-associated secretory phenotype (SASP) and release interleukin−33 (IL−33). IL−33, a member of the IL−1 family, has been shown to expand regulatory T cells (Tregs) and modulate their suppressive function, while also directly activating HSCs to promote fibrogenesis in the liver. This dual action positions IL−33 as a key mediator linking gut−derived signals to hepatic immune regulation and fibrosis in MASLD (170).

### 4.5 Single-cell and spatial omics

Advances in single-cell sequencing and spatial omics have enabled high-resolution mapping of innate–adaptive immune interactions. In complex organs like the liver, these technologies define transcriptional states, spatial localization, and functional roles of immune subsets (171, 172). scRNA-seq distinguishes innate immune cells (monocytes, macrophages, DCs) from adaptive lymphocytes (T and B cells), while pseudotime and cell–cell communication analyses predict signaling networks. In murine hepatitis models, cDC1 interacts with CD8^+^ T cells via the ALCAM-CD6 axis to enhance antigen presentation (173), and in tumor models, CXCL9-mediated chemotaxis promotes cDC1–CD8^+^ T-cell co-localization (174). Human liver studies integrating RNA sequencing (225 samples) and imaging cytometry (99 samples) revealed that myeloid cells, particularly monocyte-derived macrophages, aggregate in non-parenchymal regions correlating with MASH severity (175). Diet-induced murine MASH models further identified functional MDM subsets, including Ly6C^hi pro-inflammatory/pro-fibrotic macrophages, MoKCs, and lipid-associated macrophages(LAMs) (32). LAMs expressing TREM2, SPP1, and CD9 exhibit stage-dependent dual functions: clearing apoptotic cells and regulating lipid metabolism during progression, and promoting ECM degradation and tissue repair during regression (52).

Spatial multi-omics in 61 human liver samples (scRNA-seq of 540,216 cells, spatial transcriptomics of 47,864 spots) showed LAM expansion in MASH, localizing to centrilobular regions under hypoxia/metabolic stress, with MITF as a key regulator (24). Fibrosis-associated gene programs highlight localized crosstalk between central vein endothelial cells and HSCs. Integrative multi-omics further identified DTNA^+^ macrophages enriched in MASH, differentiating from Kupffer cells under RUNX2 regulation and interacting with activated HSCs via the RUNX2-PLG-PARD3 axis, contributing to fibrosis; DTNA also serves as a predictive biomarker (176). Overall, single-cell and spatial omics reveal that MITF-driven LAMs clear lipids and communicate with hepatocytes via HGF-MET, localize near exhausted PD-1^+^ CD8^+^ T cells in fibrotic niches, and central vein endothelial cells co-localize with HSCs to form pro-fibrotic niches. Macrophage function spans dynamic, context-dependent spectra rather than a simple pro-/anti-inflammatory dichotomy.

## 5 Treating MASLD based on innate and adaptive immunity

Growing focus centers on immune system disorders contributing to MASLD progression. Drugs such as glucagon-like peptide-1 (GLP-1) receptor agonist, farnesoid X receptor (FXR) agonist, and peroxisome proliferator-activated receptor (PPAR) agonist can not only regulate metabolic pathways but also directly modulate immune cell function, thereby influencing macrophage behavior and T cell activity. There are also some cell-based methods, such as mesenchymal stem cell (MSC) therapy and emerging chimeric antigen receptor (CAR) T cell or macrophage (CAR-M) therapy, which attempt to reprogram the inflammatory environment of the liver, inhibit fibrosis, and enhance anti-tumor immunity. In HCC caused by MASLD, immune checkpoint inhibitors (ICIs) represent a cornerstone of therapy, but their efficacy may be affected by the unique immunosuppressive environment of MASH, prompting ongoing investigations into combination strategies. These complementary methods of immunonutrition and targeted delivery of nanoparticles also provide the possibility for precise intervention. Combining these various immunotherapy methods is expected to change the development of MASLD and its related complications (**Table 3**).

**Table 3:** Immunomodulatory therapy for MASLD.

Translational stage	Therapy	Immunoregulatory & metabolic synergy mechanisms	Translational status & evidence evaluation	Reference
Approved/Guideline Recommended	THR-β agonists (e.g., Resmetirom)	Restores hepatic thyroid hormone pathways, reduces lipotoxicity-induced cellular stress, and indirectly decreases NLRP3 inflammasome activation.	First FDA-approved drug for MASH. Clinical evidence confirms its efficacy in significantly improving fibrosis and achieving MASH resolution.	(240)
Approved/Guideline Recommended	GLP-1R agonists and co-agonists (e.g., Semaglutide, Tirzepatide)	Inhibit TLR-triggered inflammation by targeting the STING signaling pathway; modulate the hepatic immune microenvironment through weight loss-dependent offloading and direct anti-inflammatory effects.	Core of the translational framework. Shifted the paradigm from isolated metabolic regulation to “systemic metabolic-immune restoration,” significantly improving MASH histological scores	(185)
Clinical Trial Stage	PPAR agonists (Lanifibranor)	Synergistically regulate hepatic fatty acid oxidation (α) and anti-inflammatory/insulin-sensitizing pathways (γ/δ), comprehensively inhibiting pro-inflammatory cytokine production.	Phase II/III clinical trials. Demonstrate potential in fibrosis improvement and MASH resolution; a focal point for multi-target immunomodulation.	(241)
Clinical Trial Stage	FXR agonists (e.g., OCA, Tropifexor)	Expressed in hepatocytes, Kupffer cells, macrophages, NKT cells, NK cells, and DCs. Regulate bile acid homeostasis and inhibit pro-inflammatory signaling pathways.	Clinical trials show fibrosis improvement, but evidence for complete MASH resolution remains inconsistent.	(242)
Clinical Trial Stage	FGF21 analogues (e.g., Efruxifermin)	Modulate lipid metabolism and reduce the release of damage-associated molecular patterns (DAMPs) linked to hepatocyte injury, thereby decreasing immune cell recruitment.	Phase IIb/III clinical trials. Show potent translational prospects in reducing hepatic inflammation and reversing fibrosis.	(243)
Clinical Trial Stage	Aspirin	Inhibits cyclooxygenase-2 (COX-2) activity and blocks the platelet-derived growth factor (PDGF) signaling pathway, exerting dual anti-inflammatory and anti-tumor effects.	Clinical exploratory stage. Initial studies show a reduction in hepatic fat content, but improvement of core MASH pathology requires large-scale validation.	(244)
Pre-clinical Stage	FFAR1/FFAR4 agonists	Promote the transition of pro-inflammatory macrophages to an anti-inflammatory phenotype and enhance the endogenous feedback of the GLP-1 axis.	Evidence limited to animal models. Show potential in ameliorating metabolic inflammation but have not entered large-scale clinical validation.	(245)
Pre-clinical Stage	MSC-EVs	Induce macrophage M2 polarization, downregulate TNF-α, IL-1β, and IL-6, secrete anti-inflammatory cytokines (IL-10), and construct an anti-fibrotic microenvironment.	Limited to experimental models. Highly promising as a cell-free therapy, but human biodistribution and safety remain to be established.	(200)
Pre-clinical Stage	CAR-T/Immune Cell Engineering	Engineered T cells recognize damaged/activated stellate cells or tumor antigens (e.g., AFP for HCC), achieving precise immune clearance.	Early exploratory stage. Primary research focus is preventing the transition from MASH to HCC; clinical applicability evidence is highly limited.	(246)
Pre-clinical Stage	Nanoparticle Delivery Systems	Targeted delivery of anti-inflammatory/anti-fibrotic agents to HSCs, precisely blocking fibrotic signaling pathways and reducing ROS levels.	Proof-of-concept stage. Aims to increase local drug concentration and reduce systemic side effects; no clinical applicability recommendations yet.	(247, 248)

### 5.1 Immunomodulatory therapies

#### 5.1.1 GLP-1 receptor agonists

GLP-1 receptor agonists represent a therapeutic strategy for MASLD and its inflammatory form, MASH. These compounds work by copying the body’s natural GLP-1 hormone to stimulate insulin secretion, suppress glucagon release, delay gastric emptying, and enhance satiety (177). The 30-amino acid incretin hormone GLP-1 acts via GLP-1R, which is expressed in the colon, pancreas, and central nervous system (178). These agonists effectively reduce hepatic lipid accumulation and inflammation; for instance, liraglutide mitigates inflammatory responses and fatty liver changes in rats fed HFD. Similarly, Exendin-4 decreases macrophage markers (CD68, F4/80) and pro-inflammatory cytokines (TNF-α, IL-1β, IL-6) in the livers of high-fat-fed mice (179). Tirzepatide, a dual agonist at GLP-1R and the glucose-dependent insulinotropic polypeptide receptor (GIPR), retains anti-inflammatory effects even in mice with reduced central GLP-1R activity, indicating multiple action pathways (180). GLP-1R activation directly modulates immune cells, as its presence on monocytes and macrophages enables it to suppress CCL2 expression in macrophages and lower circulating MCP-1 levels in MASH patients. Dual GIPR/GLP-1R agonists reduce monocyte-derived Kupffer cells and hepatic inflammatory markers. They also decrease circulating markers of liver injury. GLP-1 directly engages GLP-1R on immune cells, promoting an anti-inflammatory phenotype, such as by inhibiting the release of IL-12, IL-17A, TNF-α, and IFN-γ from intraepithelial lymphocytes. In murine hepatic tissue, GLP-1R is present on γδ T cells, where GLP-1 inhibits their inflammatory activity to reduce hepatic inflammation (181). Semaglutide treatment decreases the production of TNF-α, IL-2, CCL-2, and TGF-β in the livers of rodents on a fatty diet, while also reducing triglyceride levels and collagen accumulation (182). In human clinical trials, semaglutide has been shown to improve hepatic steatosis, insulin resistance, and visceral adiposity, although its impact on liver fibrosis remains variable. Reduced visceral and hepatic fat correlates with improved insulin sensitivity and decreased pro-inflammatory cytokines, including TNF-α, IL-1β, IL-6, IL-2, IL-17A, and IFN-γ. GLP-1 receptor agonists also exert influence over NKT cells, steer macrophages toward an M2 state, and elevate anti-inflammatory indicators like IL-10, CD163, and CD204 (183).

#### 5.1.2 FXR agonists

FXR, a BAs-activated nuclear receptor, primarily regulates bile acid, lipid, and glucose metabolism to maintain metabolic homeostasis. Although predominantly hepatic, FXR is also expressed in extrahepatic tissues such as the intestines and adrenal glands. Within the liver, FXR signaling operates in hepatocytes, cholangiocytes, and immune cells, including macrophages, NK cells, and DCs (184). FXR activation inhibits liver inflammation by dampening NF-κB signaling and enhancing anti-inflammatory factors, thereby reducing pro-inflammatory cytokine production and inflammasome activation. Preclinical MASLD studies show that pharmacological FXR activation restores hepatic metabolism and reduces inflammation. Specifically, FXR agonism promotes an anti-inflammatory phenotype in macrophages, characterized by reduced secretion of MCP-1/CCL-2 and suppression of NLRP3 inflammasome activity (185). These changes collectively alleviate hepatic steatosis, inflammation, and fibrogenesis. Furthermore, FXR activation influences HSCs through its anti-inflammatory effects, thereby preventing or reversing the progression of liver fibrosis and cirrhosis. It also significantly reduces portal pressure associated with MASH across various experimental models.

#### 5.1.3 PPARs agonists

The nuclear hormone receptor transcription factors, commonly referred to as PPARs, are classified into three distinct subtypes: PPAR-α, PPAR-γ, and PPAR-β/δ. These receptors regulate insulin sensitivity, energy balance, and glucose and lipid metabolism. Primarily localized in the liver and brown adipose tissue, PPAR-α governs fatty acid catabolism and is the target of fibrate drugs for cholesterol reduction. PPARβ/δ exists and is expressed in various tissues, and it maintains energy balance by controlling the oxidation of fat. PPAR-γ can reduce liver lipid accumulation and enhance insulin sensitivity, partly by promoting macrophages to shift from the pro-inflammatory M1 type to the anti-inflammatory M2 type. PPAR-γ agonists act in various ways, including inhibiting the production of proinflammatory cytokines and regulating the secretion of adipokines (186). In MASLD, PPAR dysfunction is closely linked to lipid metabolism disorder, insulin resistance, inflammation, and liver fibrosis, making these receptors an important therapeutic target. Studies have confirmed that up-regulation of PPAR-γ in macrophages enhances fatty acid oxidation, reduces ROS production, and inhibits inflammation, while down-regulation or specific knockout will aggravate liver inflammatory damage. PPAR-γ can also play a protective role by activating the Keap1-Nrf2 pathway (187–189). PPAR agonist lanifibranor activates all three subtypes at the same time (190). Studies on liver cells confirmed that this drug improved steatohepatitis in mice fed a CDAA-HFD diet. Short-term treatment reduces liver macrophage infiltration and helps to reduce fat accumulation and inflammation. Other drugs, such as the PPARα/δ dual agonist elafibranor, restore intestinal integrity and reduce liver inflammation by regulating the intestinal-liver axis communication. PPAR-γ antagonist GW9662 can delay the progression of MASLD by down-regulating the TLR4/NF-κB signaling pathway, while PPARδ inhibitor GW0742 can also reduce inflammation by inhibiting the TLR4/MyD88/NF-κB signaling pathway. PPARs play a key role in liver pathology by coordinating and regulating metabolism, inflammation, and fibrosis (191).

### 5.2 Cell therapy

#### 5.2.1 MSCs therapy

MSCs are pluripotent stem cells that can differentiate, self-renew, and have low immunogenicity, making them a popular choice for cell therapy. Their main therapeutic effect is not to directly differentiate into hepatocytes, but to release cytokines and exosomes through paracrine signaling to resist inflammation, fibrosis, and promote regeneration (192). These cells exhibit context-dependent immunomodulation: in highly pro-inflammatory environments with IFN-γ and TNF-α or upon TLR3 stimulation, they suppress immune responses by inhibiting lymphocyte proliferation, enhancing regulatory T cell generation, and promoting M2 macrophage polarization. Extracellular vesicles derived from MSCs, particularly exosomes, retain the therapeutic properties of the parent cells while offering improved safety, low immunogenicity, and no tumorigenic risk (193). In experimental models of MASH, MSCs and their exosomes demonstrate significant therapeutic efficacy. Human umbilical cord MSC-derived exosomes enhance the activity of liver cell lipid metabolism genes, including SREBP-1c and PPAR-α, which in turn lessen liver fat buildup, oxidative stress, and inflammation (194). The delivery of p-STAT3 promotes arginase-1 transcription and induces an M2 anti-inflammatory phenotype, as well as the suppression of excessive NF-κB activation via the LPS/TLR4/MyD88 pathway (195). MSC transplantation can also reverse liver fibrosis by decreasing hepatic infiltration of CD11b+ and Gr-1+ cells, increasing the Treg/Th17 ratio, and downregulating IL-17-associated signaling pathways such as STAT3 and TGF-β (196).

Genetic modification and combination therapies optimize the therapeutic effects of MSCs. IL-12-engineered MSCs enhance antitumor immunity against hepatocellular carcinoma in MASLD by activating NK and CD8+ T cells. MSC-liraglutide combinations improve metabolic parameters and liver repair while suppressing TLR4/NF-κB-mediated inflammation. Bone marrow MSCs reduce liver fibrosis in obesity by modulating inflammatory and fibrotic mediators (197). Therefore, to develop a safe and effective treatment for liver diseases, we must deeply understand the specific regulatory mechanisms of MSCs and their exosomes in a specific immune microenvironment.

#### 5.2.2 CAR cell therapy

The core of CAR-T cell therapy is the modified chimeric CAR. Its extracellular part, single-chain variable fragment (ScFv), can specifically recognize the antigen on the tumor surface, while intracellular signaling regions, such as CD3ζ, can activate immune cells. This design allows CAR-T cells to directly target and kill cancer cells without relying on MHC antigen presentation, thus countering the key mechanism of tumor immune escape (198). CAR-T therapy is an advanced cellular immunotherapy. T cells are genetically modified to accurately target specific cancer antigens. It combines the cytotoxicity of T cells with the accuracy of antibodies to create a highly targeted treatment. The process involves introducing CAR-encoding genes into T cells, expanding them *in vitro*, cultivating potent effector cells and memory lymphocytes, and then reintroducing them to patients, allowing them to proliferate and exert a therapeutic effect (199). Therefore, CAR-T cells are often referred to as “living drugs” because they can reproduce, become lasting memory cells, and trigger specialized and long-term anti-tumor immune response (200). In the study of HCC, scientists primarily focus on Glypican-3(GPC-3), alpha-fetoprotein (AFP), and tumor-associated antigen Carcinoma-1 (NY-ESO-1) (201). In the MASH mouse model, CAR-T cells targeting uPAR reduced fibrosis and eliminated senescent cells (202). A new generation of technology, such as bispecific CAR-T cells targeting both GPC3 and ASGR1, has shown a stronger anti-tumor effect and less off-target toxicity in a preclinical *in situ* HCC model, which provides a new direction for solid tumor treatment.

Beyond CAR-T, macrophage-based therapies also hold potential. Preclinical studies confirm that infusing ex vivo-differentiated macrophages can alleviate liver fibrosis in mice. A preliminary clinical trial suggests that reinfusing autologous, ex vivo-induced macrophages with a repair phenotype is safe and feasible and correlates with a trend toward improved liver function scores (203). However, the therapeutic efficacy remains uncertain, challenged by issues of cellular retention, phenotypic stability, and reliable cell sources. To address these, researchers are exploring induced pluripotent stem cells for generating CAR-M. CAR-M is developing very fast. For example, the newly developed switchable CAR-M can start anti-inflammatory and tissue repair programs according to inflammatory signals, which provides a new method for inflammatory diseases. In cancer treatment, the traditional CAR-M can selectively eat tumor cells, turn them into M1 type, activate related signal pathways, and enhance the recruitment and activation of T cells. Animal experiments show that in the orthotopic liver cancer model, CAR-M alone can make the tumor disappear completely (204). Although the prospect is good, there are still many challenges in the treatment of MAFLD-HCC with cell therapy, such as how to standardize cell preparation, optimize treatment time and dose, and evaluate long-term safety.

### 5.3 Other immunotherapies

#### 5.3.1 ICIs

Although immunotherapy, especially Programmed death-1/programmed death-ligand 1(PD-1/PD-L1) inhibitors, has revolutionized the treatment of virus-related liver cancer, its effect in MASLD/MASH-related HCC remains unstable. This difference is partly due to the unique liver immune microenvironment of MASLD/MASH. MASH depletes anti-tumor CD8+ T cells, thereby increasing exhausted PD-1+ variants, potentially compromising anti-tumor immunity. The MASH-HCC microenvironment uniquely features interactions between exhausted PD-1+ CD8+ T cells, PD-L1+/ICOS+ myeloid suppressors, and tumor-associated macrophages—rarely observed in viral HCC (113). These differences in mechanism partly explain why the efficacy of ICIs alone is limited. Clinical retrospective analysis also found that the disease control rate of liver cancer patients with MASH-related cirrhosis may be lower than that of patients without MASH (205). However, ICIs are still the basic treatment for liver cancer. PD-1/PD-L1 pathway is crucial for tumor immune escape, and drugs that block it (such as nivolumab and pembrolizumab) are effective in clinical trials, which is a common choice for advanced liver cancer (206). Nowadays, more and more attention is paid to combination therapy to improve the effect. For instance, the combination of atezolizumab (anti-PD-L1) and bevacizumab (anti-VEGF) is more effective for unresectable liver cancer. Nivolumab in combination with ipilimumab (anti-CTLA-4) has been approved for advanced liver cancer. In the CheckMate-040 trial, the objective response rate reached 32%, and the median overall survival time was 22.8 months. Phase III trial also showed that the STRIDE regimen (tremelimumab plus durvalumab) can improve the overall survival and safety more than sorafenib (207). However, in MASLD/MASH-related HCC, PD-1/PD-L1 targeting drugs alone may pose special risks. Animal studies have found that inhibiting PD-1 will aggravate the liver injury caused by CD8+ T cells in the MASH environment, which may worsen HCC instead, and in some experiments, it did not significantly reduce tumor size. Clinical data also show that the survival rate of patients with MASLD-related HCC who use PD-1/PD-L1 inhibitors is lower (208). Therefore, scientists are currently studying to transform the immunosuppression microenvironment, such as the myeloid-modifying cells by inhibiting Trem2, combining anti-fibrosis drugs (like angiotensin II receptor antagonist losartan) with immunotherapy to reduce fibrosis and increase CD8+ T cell infiltration, and targeting other molecules such as METTL3 to activate anti-tumor immunity (112, 209, 210). Lifestyle interventions such as exercise have been shown to improve MASH’s condition and reduce the accumulation of PD-1+ CD8+ T cells. Basic research also found that certain factors, including TRIM21, can influence the development of MASH-related HCC by regulating the expression of PD-1/PD-L1 (211).

#### 5.3.2 Immunonutrition

Immunonutrition employs specific nutrients to prevent and treat infection, inflammation, or damage-related diseases. Immunonutrients are substances that directly or indirectly modulate the immune response via antioxidant activity, immune cell regulation, and inflammatory pathway control (212). There are amino acids such as cysteine and arginine, polyphenols such as flavonoids and phenolic acids, vitamins C and D, trace elements such as zinc, fatty acids, and nucleotides. Polyphenols play a role mainly by scavenging reactive oxygen, improving lipid metabolism, and reducing systemic inflammation. In human studies, the Mediterranean diet (MD), which has been supported by research for a long time, is rich in these immunonutrients and can provide antioxidant and anti-inflammatory effects at the same time. This dietary pattern can reduce the fat content in the liver and regulate the intestinal flora through dietary fiber, prebiotics, and omega-3 fatty acids. It can reduce pro-inflammatory rumen bacteria and increase short-chain fatty acid producers such as Clostridium leptum and Eubacterium rectale (213). Supplementing mice with Docosahexaenoic acid (DHA) and eicosapentaenoic acid (EPA) mitigates the adverse effects of high-fat diets, reducing liver fat and inflammatory markers, and limiting protein activity in fat cell development (214). Vitamin D, another multifunctional immunonutrient, participates broadly in immune-inflammatory and metabolic processes beyond calcium homeostasis. Its active form acts through the vitamin D receptor, a key axis influencing metabolic organs, including the liver. Human studies indicate that hepatic vitamin D receptor (VDR) expression inversely correlates with steatosis severity and inflammation. As an immunomodulator, vitamin D suppresses Th1 and Th17 responses while promoting Th2 and regulatory T cell functions, thus maintaining immune tolerance in microenvironments such as the gut (215). Stimulating VDR activity in liver macrophages reduces inflammation, fat accumulation, and impaired glucose regulation. Seven randomized controlled trials targeting patients with solid tumors undergoing chemotherapy (total n = 521) demonstrated heterogeneity in cancer types, immunonutrient formulations, administration routes, and control group design, with intervention durations ranging from 4 to 14 weeks. None of the studies reported absolute infection counts, and only three reported adverse events such as febrile neutropenia and pneumonia; some studies observed reductions in CRP and TNF-α. The current evidence is insufficient to conclusively determine the effect of immunonutrition on infectious complications in adult chemotherapy patients, highlighting the urgent need for high-quality studies that consider specific cancer types, malnutrition status, dosage, timing, and duration (216). Muscle atrophy is often associated with dysregulated inflammatory cytokines, which may influence the efficacy of immunotherapy. However, these findings may not fully reflect the underlying biological reality. Although factors such as sex can affect the accumulation of subcutaneous (SAT) and visceral adipose tissue (VAT), their individual contributions are difficult to disentangle due to high intercorrelation. One approach to better understand these associations is further stratification based on individual VAT and SAT levels in clinical trials. In this context, the use of fixed-dose regimens has raised additional concerns. Such approaches do not account for body composition, which is particularly problematic in patients with high fat mass or reduced muscle mass. Given their potential roles in drug distribution and metabolism, uniform dosing may not maximize therapeutic efficacy (217). The American Society for Parenteral and Enteral Nutrition (ASPEN) recommends the use of immunomodulating enteral formulations in major elective surgery, trauma, burns, and mechanically ventilated critically ill patients; however, caution is advised in cases of severe sepsis. No significant differences in severe adverse events or length of hospital stay were observed between patients receiving immunonutrition versus standard nutrition (218).Some patients are unable to reach target infusion rates due to gastrointestinal intolerance, limiting the effective delivery of immunonutrients as part of nutritional regimens (219).Although multiple studies have reported favorable outcomes of immunonutrition, including reduced infectious complications, shorter duration of mechanical ventilation, and decreased mortality, not all studies demonstrated positive results, and a few suggested potential harm, particularly in critically ill patients with concomitant sepsis, indicating a risk of adverse reactions (220).The optimal composition of immunonutrition remains undetermined and may vary according to patient comorbidities and planned surgical procedures. Prior to initiating such interventions, risk stratification based on markers of nutritional deficiency (e.g., imaging-assessed sarcopenia, Glasgow Prognostic Score, Prognostic Nutritional Index, or methylated arginine assessment) is warranted. Finally, well-designed randomized controlled trials are needed to stratify patients appropriately and determine the optimal timing, composition, and duration of immunonutrition (221).

#### 5.3.3 Nanoparticle delivery systems

Nanomedicine provides an innovative treatment strategy for liver diseases by enabling precise targeting, improving drug metabolism, and reducing systemic toxicity. Its main advantage is that it can specifically deliver drugs to key pathogenic cells, such as activated HSCs in fibrosis, inflammatory hepatocytes in MASLD, or tumor cells in liver cancer (222). Nanoformulations achieve this cell-selective targeting by utilizing specific cell surface receptors (such as retinoic acid receptors, platelet-derived growth factor (PDGF) receptors, or integrins). For example, lipid nanoparticles loaded with siRNA targeting heat shock protein 47 (HSP47) and targeting HSCs with retinol-binding protein have entered Phase II clinical trial in patients with compensated MASH cirrhosis (223). Achieving true specificity for HSCs is challenging, as some designed nanoparticles may be non-specifically phagocytosed by macrophages. Future success depends on a deeper understanding of HSCs and myofibroblast subpopulations and their activation pathways, which will enable more precise anti-fibrotic therapies. Nanotechnology can also directly interfere with collagen synthesis by delivering either activating or inhibitory molecules such as microRNAs. In MASLD, nanodelivery systems are used to modulate key metabolic pathways. Polymeric nanoparticles delivering mammalian Ste20-like kinase 1(MST1), for instance, can activate the AMP-activated protein kinase/sterol regulatory element-binding protein 1c(AMPK/SREBP-1c) axis to improve hepatic insulin sensitivity and reduce steatosis (224). Research has also revealed complex interactions between nanomaterials and biological systems. Polystyrene nanoparticles, for example, disrupt macrophage activity and lipid metabolism by interfering with the PPAR pathway. For interventions targeting the gut-liver axis, researchers have developed multiple responsive nanosystems. pH- and microbiome-dual-responsive nanoparticles can deliver nitrochloride, improving metabolic markers, reducing inflammation, and modulating gut microbiota in MASLD mouse models (225). Other methods will use nanocapsules to slowly release hydrogen, which can improve metabolism and increase the abundance of bacteria such as *Akkermansia muciniphila.* FT@XBP1 nanoparticles, for example, can be used to reduce endoplasmic reticulum stress, thereby repairing the intestinal barrier and restoring normal gut microbiota. A Nano-Amorphous Formula of Atorvastatin containing prebiotics demonstrates superior efficacy than conventional drugs, and it can be better absorbed by the body, so it has a stronger anti-inflammatory effect and is healthier for the liver. In the treatment of hepatocellular carcinoma, nanotechnology will exploit specific characteristics of the tumor microenvironment, such as hypoxia, many receptors, acidic environment, and easier infiltration of blood vessels, to achieve accurate and controllable drug delivery (226–228). The design of nanocarriers is crucial, typically involving a core, a targeting unit, and a stimulus response unit. There are various materials used as nanocarriers for hepatocellular carcinoma, including organic dendrimers, lipid nanoparticles, nanogels, and inorganic materials such as metal nanoparticles, quantum dots, and carbon-based structures. Targeting ligands include small molecules such as glycyrrhetinic acid and folic acid, proteins such as transferrin and GPC3, as well as antibodies, aptamers, and polypeptides. For example, folate-modified prodrug nanoparticles can simultaneously target folate receptors and asialoglycoprotein receptors overexpressed in hepatocellular carcinoma cells, thereby improving therapeutic efficacy (229). Scientists have also developed various functional nanoparticles, which can be directly used to fight tumors. For example, platinum nanoparticles modified with peptides can induce apoptosis, while mPEG-PLGA-PLL loaded with arsenic trioxide can enhance the anti-tumor effect by up-regulating GSD ME-N. Carbon quantum dots have two uses: they can mark cells and inhibit tumor growth by inducing autophagy. Inorganic nanoclusters like ZnS@BSA can activate the cGAS/STING pathway and promote the activation of CD8+ T cells (230–232). However, despite good prospects, the clinical application of nano-drugs still faces some challenges, such as stability, shelf life, potential immunogenicity, and complex quality control issues.

## 6 Conclusion

The pathogenesis of MASLD involves the complex and changing interaction between innate and adaptive immune cells in the damaged liver environment. This review explains how KCs, neutrophils, NKT cells, DCs, and resident immune cells, such as T cells and B cells, can jointly promote disease development through continuous bidirectional signaling. This intercellular dialogue occurs through several mechanisms: direct receptor-ligand binding, cytokine signaling cascades, and the complement system. In addition, the gut-liver axis affects this immune metabolic network, as microbial by-products and a damaged intestinal barrier will continue to cause liver inflammation and fibrosis. These integrated pathways together form a self-reinforcing cycle that promotes the development of diseases from initial fat accumulation to advanced liver injury, cirrhosis, and HCC.

Scientists have recently discovered that immune and metabolic processes interact with each other, presenting new opportunities for treating diseases. Some drugs can affect nuclear receptors and incretin signaling. They can not only regulate metabolism, but also control the immune system. Cell therapy, such as mesenchymal stromal cells, CAR-modified T cells, or macrophages, may help modify the immune environment of the liver, reduce fibrosis, and enhance the ability to fight tumors. However, MASLD-related HCC often weakens the immune system, which makes it more difficult for drugs such as ICIs to work, so a treatment strategy that can solve metabolic problems and immune escape at the same time is needed. Advanced methods such as immunonutrition and nanocarrier systems may offer targeted treatments, but they need to be carefully studied before practical application. Future research should focus on elucidating the unique regulatory pathways of cells and developing more potent multi-target immunotherapies to address MASLD and its associated complications. A systematic study of how immunity and metabolism interact during disease development will lay an important foundation for designing precise treatment strategies for different stages of MASLD.

## Glossary

MASLD: metabolic dysfunction-associated steatotic liver disease
MASH: metabolic dysfunction-associated steatohepatitis
HCC: hepatocellular carcinoma
IL-10: interleukin-10
TGF-β: transforming growth factor beta
IL-12: interleukin-12
IL-23: interleukin-23
KCs: Kupffer cells
ECM: extracellular matrix
IL-6: interleukin-6
NASH: non-alcoholic steatohepatitis
FFAs: free fatty acids
IL-1β: interleukin-1β
TNF-α: tumor necrosis factor-alpha
NETs: neutrophil extracellular traps
HSCs: hepatic stellate cells
MPO-DNA: myeloperoxidase-DNA
miR-223: MicroRNA-223
MMP9: matrix metalloproteinase-9
HFD: high-fat diet
ROS: reactive oxygen species
NKT: natural killer T
TCRs: T cell receptors
iNKT: invariant natural killer T
Hh: Hedgehog
NF-κB: nuclear factor kappa-B
Tim-3: T cell Ig and mucin domain 3
LTβR: lymphotoxin β-receptor
TNFSF14: tumor necrosis factor superfamily member 14
LSECs: liver sinusoidal endothelial cells
ICAM-1: intercellular adhesion molecule-1
VCAM-1: vascular cell adhesion molecule-1
IFN-γ: interferon-γ
VAP-1: vascular adhesion protein-1
IL-4: interleukin-4
DCs: dendritic cells
DCregs: regulatory dendritic cells
CDP: dendritic cell progenitor
cDC1s: type 1 dendritic cells
cDC2s: type 2 dendritic cells
pDCs: plasmacytoid dendritic cells
Tregs: regulatory T cells
Bregs: regulatory B cells
RORγt: receptor-related orphan receptor gamma-t
ODC1: ornithine decarboxylase 1
HFHC: high-fat high-choline
ALT: alanine aminotransferase
AST: aspartate aminotransferase
α-SMA: α-smooth muscle actin
MCD: methionine–choline-deficient
ISGs: interferon-stimulated genes
IL-11: interleukin-11
MAC: membrane attack complex
MBL: mannose-binding lectin
MAMPs: microbe-associated molecular patterns
LPS: lipopolysaccharide
NLRs: NOD-like receptors
SCFAs: short-chain fatty acids
PAMPs: pathogen-associated molecular patterns
Bas: bile acids
GVB: gut vascular barrier
GLP-1: glucagon-like peptide-1
PPAR: peroxisome proliferator-activated receptor
FXR: farnesoid X receptor
MSC: mesenchymal stem cell
GIPR: glucose-dependent insulinotropic polypeptide receptor
ICIs: immune checkpoint inhibitors
GPC-3: Glypican-3
AFP: alpha-fetoprotein
CAR: chimeric antigen receptor
ScFv: single-chain variable fragment
EPA: eicosapentaenoic acid
PDGF: platelet-derived growth factor
HSP47: heat shock protein 47
MST1: mammalian Ste20-like kinase 1
AMPK/SREBP-1c: AMP-activated protein kinase/sterol regulatory element-binding protein 1c
MDSCs: myeloid-derived suppressor cells
TAMs: tumor-associated macrophages
LAMs: lipid-associated macrophages
LTA: lipoteichoic acid
SASP: senescence-associated secretory phenotype
